# Long non-coding RNA MIR200CHG promotes breast cancer proliferation, invasion, and drug resistance by interacting with and stabilizing YB-1

**DOI:** 10.1038/s41523-021-00293-x

**Published:** 2021-07-16

**Authors:** Li Tang, Da Wei, Xinyu Xu, Xuelian Mao, Dongping Mo, Linping Yan, Weiguo Xu, Feng Yan

**Affiliations:** 1 Department of Clinical Laboratory, Jiangsu Cancer Hospital & Jiangsu Institute of Cancer Research & the Affiliated Cancer Hospital of Nanjing Medical University, Nanjing, P. R. China; 2 Department of Surgery, Jiangsu Cancer Hospital & Jiangsu Institute of Cancer Research & the Affiliated Cancer Hospital of Nanjing Medical University, Nanjing, P. R. China; 3 Department of Pathology, Jiangsu Cancer Hospital & Jiangsu Institute of Cancer Research & the Affiliated Cancer Hospital of Nanjing Medical University, Nanjing, P. R. China

**Keywords:** Breast cancer, Breast cancer

## Abstract

Long non-coding RNAs (lncRNA) have been identified as key regulators of tumorigenesis and development. We aim to explore the biological functions and molecular mechanisms of lncRNA MIR200CHG in breast cancer. We found that MIR200CHG is highly expressed in breast cancer tissues and is related to the tumor size and histopathological grade. In vitro and in vivo experiments confirmed that MIR200CHG can promote breast cancer proliferation, invasion, and drug resistance. MIR200CHG directly binds to the transcription factor Y-box binding protein-1 (YB-1), and inhibits its ubiquitination and degradation. MIR200CHG regulates YB-1 phosphorylation at serine 102, thereby affecting the expression of genes related to tumor cell proliferation, apoptosis, invasion, and drug resistance. Additionally, MIR200CHG partially affects the expression of miR-200c/141-3p encoded by its intron region. Therefore, MIR200CHG can promote the proliferation, invasion, and drug resistance of breast cancer by interacting with and stabilizing YB-1, and has the potential to become a target for breast cancer treatment.

## Introduction

Breast cancer is the most common malignant heterogeneous tumor in women and is the main cause of cancer deaths among women^1–3^. Many scientists are committed to the development of breast cancer molecular targeted therapies and individualized clinical treatment, which have effectively reduced the mortality rate of breast cancer^4–6^. Studies have confirmed that long non-coding RNA (lncRNA) can be used as a tumor suppressor or oncogene to participate in the development and regulation of breast cancer, and has the potential to become a target for breast cancer detection and treatment^7–9^.

LncRNA is defined as an RNA transcript greater than 200 nt, which has no obvious protein coding potential, but can participate in chromatin remodeling and modification, and signal transduction regulation^10^. Some lncRNAs can act as host genes for microRNAs (miRNAs) with a length of about 22 nt, and can form independent functional lncRNAs and miRNAs^11,12^. We used lncRNA microarrays to compare the lncRNA expression between cancer tissues and adjacent non-tumor tissues in six breast cancer patients, and found that lncRNA U47924.27 was significantly upregulated in cancer patients^13^. *U47924.27* is annotated as *homo* sapiens MIR200C and MIR141 host gene (MIR200CHG; Accession number: NR_135032.1), and its intron region encodes miR-200c and miR-141. miR-200c/141 belong to the miR-200s family, which mainly regulates the expression of target genes after transcription by promoting mRNA degradation or inhibiting translation, and its functions are dependent on the tumor type and cell background^14^. Although the role of miR-200c/141 has been widely characterized, the nature of MIR200CHG is still completely unknown.

In this study, we show the differences between cancer tissues and adjacent non-tumor tissues in MIR200CHG expression and the mechanism of the proliferation, invasion, and drug resistance in breast cancer cell promoted by MIR200CHG.

## Results

### MIR200CHG is upregulated in breast cancer and is related to the tumor size and histopathological grade

**Fig. 1:**
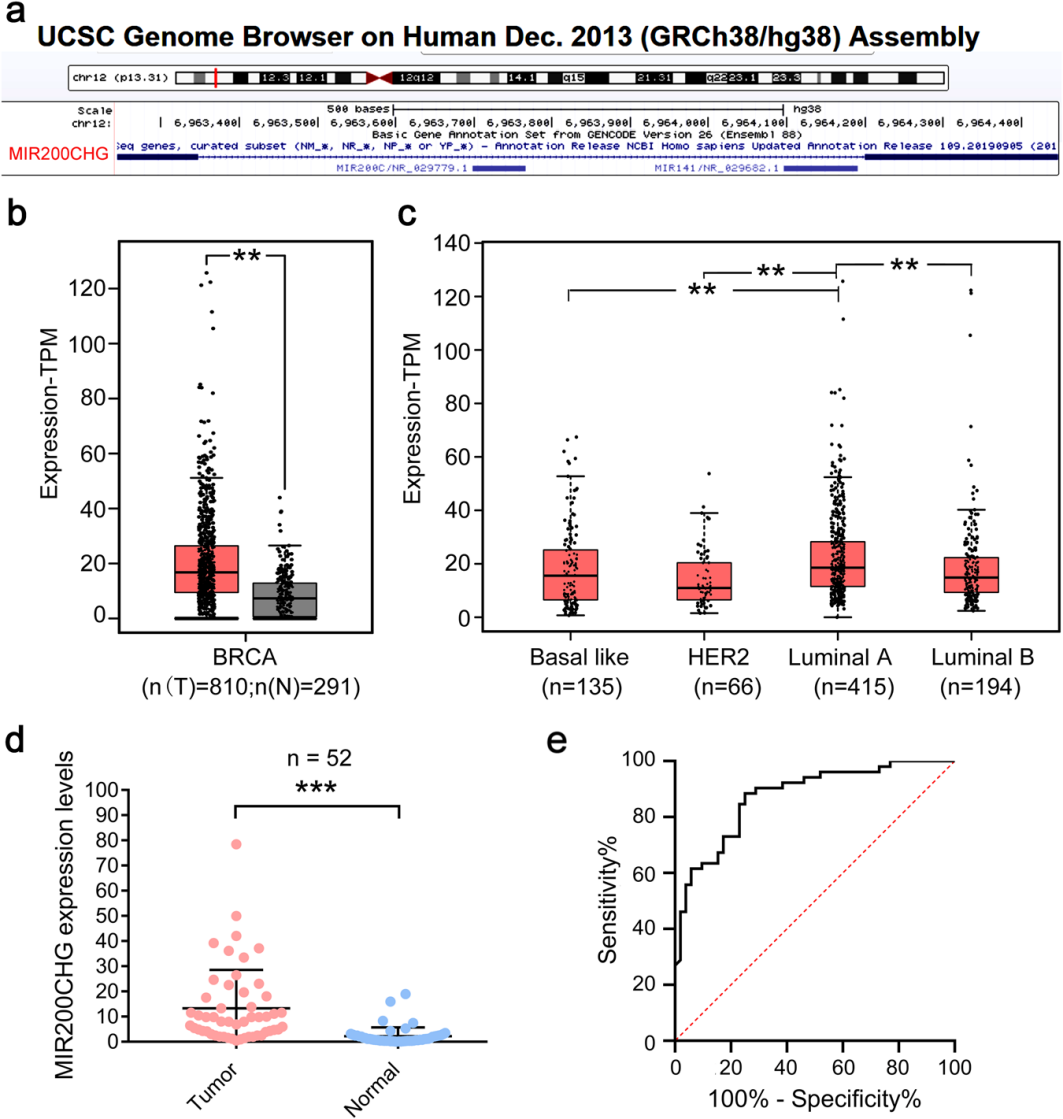
MIR200CHG is upregulated in breast cancer tissues. **a** Gene structure of MIR200CHG. **b**, **c** The expression of MIR200CHG in breast cancer and its subtypes in The Cancer Genome Atlas was analyzed by GEPIA2. N, non-tumor tissue, gray. T, tumor tissue, red. **P < 0.01. Boxplots represent medians (center line) and interquartile range (IQR; box), and whiskers represent the maximum and minimum values within 1.5 times the IQR from the edge of the box. **d** qRT-PCR was used to analyze the expression level of MIR200CHG in breast cancer tissues and adjacent non-tumor tissues, *n* = 52. GAPDH was used as an internal control. Dot plot of the individual data points, with a horizontal line at the arithmetic mean and error bars showing plus and minus one standard deviation. The data were analyzed by two-tailed unpaired *T* test. ***P < 0.001. **e** Receiver operating characteristic curve analysis based on the level of MIR200CHG in 52 analyzed breast cancer tissues.

LncRNA microarray analysis reveals that lncRNA MIR200CHG is highly expressed in breast cancer tissues compared to the adjacent normal tissues (fold change = 17.042, *P* = 0.022) (Supplementary Fig. 1a). The UCSC genome browser (http://genome.ucsc.edu/) shows that the *MIR200CHG* gene is located on chromosome 12p13 and contains two exons and one intron. These two exons can be spliced to form a non-coding transcript MIR200CHG (NR_135032) with a length of 366 nt and a poly A tail (Fig. 1a). MIR200CHG has low conservation among species (PhyloCSF score = −128.3372, cutoff: 60.7876). Coding-Potential Assessment Tool (http://lilab.research.bcm.edu/cpat/index.php) predicts that MIR200CHG has a low coding probability (coding probability = 0.0208, cutoff: 0.364). ORF finder (https://www.ncbi.nlm.nih.gov/orffinder/) discovers some shorter open reading frames on MIR200CHG, but BLAST (https://blast.ncbi.nlm.nih.gov/Blast.cgi) shows that there is no obvious match between the predicted short peptide sequence and the known protein amino acid sequence. These analyses indicate that MIR200CHG has typical characteristics of non-coding transcripts. We use GEPIA2 (http://gepia2.cancer-pku.cn/#index) to analyze The Cancer Genome Atlas (TCGA) data and find that MIR200CHG is upregulated in breast cancer (Fig. 1b), and there are differences in expression between the molecular subtypes (Fig. 1c). Although MIR200CHG is also upregulated in several other cancers (Supplementary Fig. 1b), it is downregulated in kidney renal clear cell carcinoma (KIRP), kidney renal papillary cell carcinoma (KIRC), and skin cutaneous melanoma (SKCM) (Supplementary Fig. 1c), indicating that MIR200CHG may function in a context-dependent manner. We use qRT-PCR to detect MIR200CHG in 52 pairs of breast cancer and adjacent normal tissues (Fig. 1d). The ROC curve shows that MIR200CHG has a high diagnostic efficiency (AUC = 0.875, 95% confidence interval, 0.809–0.946, *P* < 0.001) (Fig. 1e). On analyzing the clinicopathological data, we find that the expression level of MIR200CHG is correlated with the tumor size (*P* = 0.035) and histopathological grade (*P* = 0.039) (Table 1).

**Table 1:** Relationship between MIR200CHG expression and pathological features in breast cancer patients.

Variable	Number	Relative expression of MIR200CHG		
		High	Low	*P* value
Age (years)				0.782
≤50	23	8	15	
>50	29	12	17	
Menopause				0.178
Yes	29	14	15	
No	23	6	17	
Tumor size				0.035^a^
≤2 cm	30	7	23	
>2 cm	22	13	9	
Grade				0.039^a^
G1	14	2	12	
G2–3	38	20	18	
LN metastasis				0.182
Yes	31	14	17	
No	21	5	16	
Distant metastasis				0.135
Yes	15	6	9	
No	37	12	25	

Mann–Whitney *U* test for the comparison between two groups.

*LN* lymph node.

^a^*P* < 0.05.

### MIR200CHG promotes the malignant behavior of the breast cancer cells in vitro

**Fig. 2:**
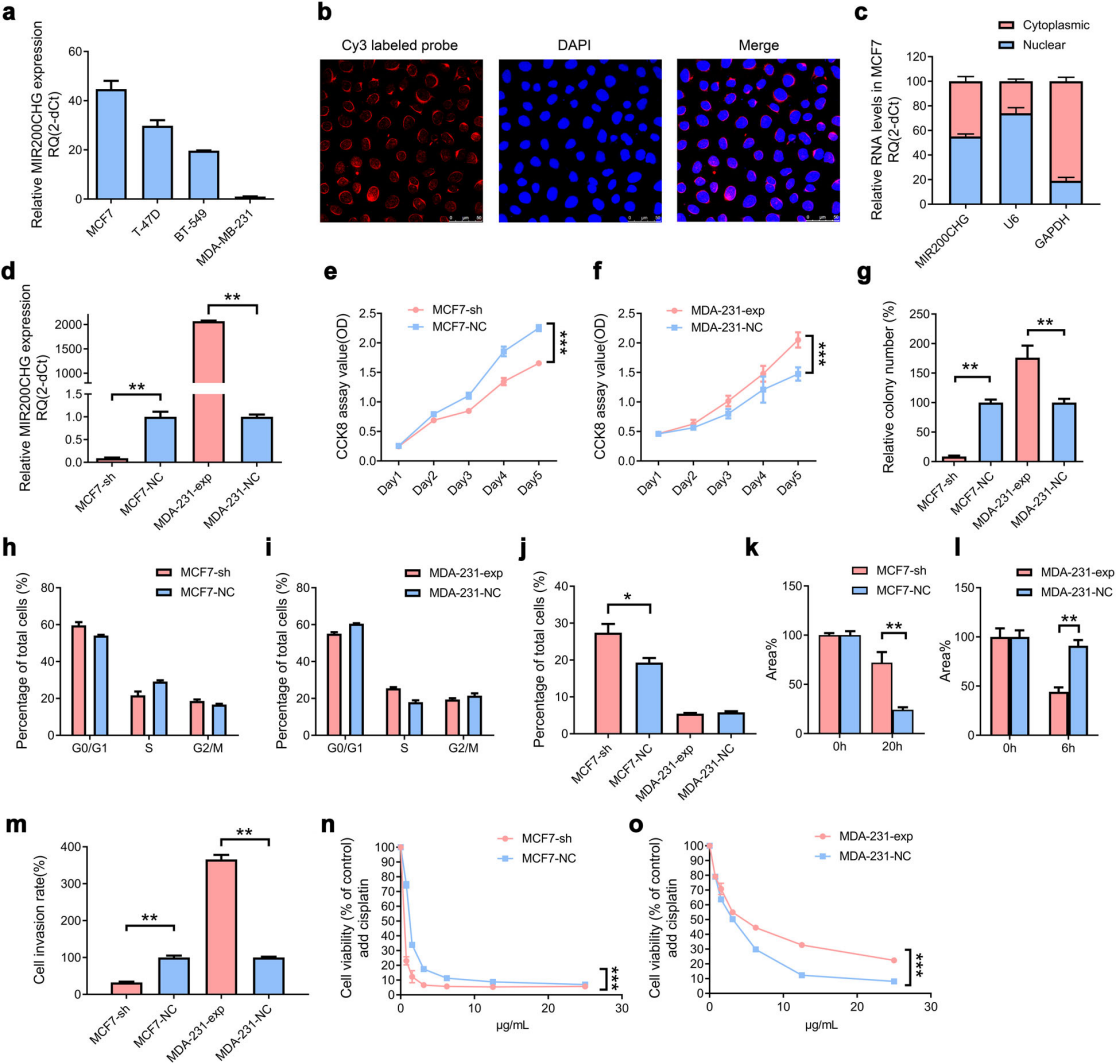
MIR200CHG promotes breast cancer cell proliferation, invasion, and drug resistance in vitro. **a** qRT-PCR analysis of the expression levels of MIR200CHG in breast cancer cell lines (MCF7, T-47D, BT-549, MDA-MB-231). Experiments were performed at least three times in triplicate, and data are represented as mean ± SEM. MDA-MB-231 cells were used as a reference to calculate the relative expression of MIR200CHG. **b** Representative confocal microscope images of MIR200CHG distribution in MCF7 cells with immunofluorescence staining. (×630, oil lens). **c** qRT-PCR analysis of the distribution of MIR200CHG in the nucleus and cytoplasm of MCF7 cells. U6 was used as an internal reference for the quality of nuclear RNA extracted, and GAPDH was used as an internal reference for the quality of cytoplasmic RNA extraction. Data were confirmed in three independent experiments and are represented as mean ± SEM in triplicates. **d** Effects of knocking down or overexpressing MIR200CHG in MCF7 or MDA-MB-231 cells using the lentiviral system were detected by qRT-PCR. **e**, **f** CCK-8 assays showing the effects of MIR200CHG on breast cancer cell proliferation. **g** Effects of MIR200CHG on colony formation in breast cancer cells. **h**, **i** FACS analysis of the effect of MIR200CHG knockdown or overexpression on the cell cycle. **j** FACS analysis of the effect of MIR200CHG knockdown or overexpression on the cell apoptosis. The right quadrant shows the percentage of apoptotic cells. MCF7-sh group is about 27.40%, MCF7-NC group is about 19.31%; MDA-231-exp group is about 5.46%, MDA-231-NC is about 5.82%. **k**, **l** Wound healing experiment demonstrating the effects of MIR200CHG on the migration ability of breast cancer cells. **m** Transwell assay showing the effects of MIR200CHG on the invasion ability of breast cancer cells. MCF7 cells were incubated for 24 h, and MDA-MB-231 cells were incubated for 10 h. **n**, **o** CCK-8 assays demonstrating the effects of MIR200CHG expression on the sensitivity of breast cancer cells to cisplatin. Experiments in (**d**–**o**) were performed at least three times in triplicate, and data are represented as mean ± SEM. Two-way ANOVA were used to analyze the data in (**e**, **f**, **n**, **o**) and two-tailed *T* test was used to analyze the data in (**d**, **g**–**m**). *P < 0.05, **P < 0.01, ***P < 0.001.

We test several breast cancer cell lines (MCF7, T47D, BT-549, and MDA-MB-231) and find that MIR200CHG expression is higher in MCF7 cells and lower in MDA-MB-231 cells (Fig. 2a). FISH experiments reveal that MIR200CHG is distributed in the nucleus and cytoplasm, especially around the nucleus (Fig. 2b). qRT-PCR results show that about 55.1% and 44.9% of MIR200CHG are distributed in the nucleus and cytoplasm, respectively (Fig. 2c). Next, we knock down MIR200CHG in MCF7 cells (downregulated to 9.04%), and overexpress MIR200CHG in MDA-MB-231 cells (upregulated to 2066.74%) to perform gain and loss of function experiments (Fig. 2d). Knockdown of MIR200CHG expression reduce the proliferation of MCF7 cells, while overexpression of MIR200CHG increase the proliferation of MDA-MB-231 cells (Fig. 2e, f). Colony formation experiments confirm that compared with the control group, knockdown of MIR200CHG reduce the MCF7 cell colony formation rate to 8.62%, while overexpression of MIR200CHG increase the rate of MDA-MB-231 cell colony formation to 176.17% (Fig. 2g, Supplementary Fig. 2a). Flow cytometry analysis show that compared with the negative control group, the cell cycle distribution of MCF7-sh and MDA-231-exp does not change significantly (Fig. 2h, i, Supplementary Fig. 2b). Apoptosis analysis shows that knockdown of MIR200CHG increased the apoptosis rate in MCF7 cells, while overexpression of MIR200CHG does not significantly change the apoptosis rate in MDA-MB-231 cells (Fig. 2j, Supplementary Fig. 2c). Wound healing experiments show that the average open wound area of MCF7-sh cells (incubated for 20 h) is about 72.1%, and that of negative control cells is about 24.2%. The average open wound area of MDA-231-exp cells (incubated for 6 h) is about 44.2%, and that of the negative control cells is about 91.0% (Fig. 2k, l, Supplementary Fig. 2d). In the cell invasion experiments, the number of MCF7-sh cells passing through the Matrigel pores is 32.5% of the negative control group, while the number of MDA-231-exp cells reaches 365.8% (Fig. 2m, Supplementary Fig. 2e). Furthermore, on conducting cisplatin drug sensitivity experiments, knockdown of MIR200CHG significantly decreases the IC_50_ of MCF7 cells from 1.25 ± 0.08 μg/mL to 0.12 ± 0.05 μg/mL, while overexpression of MIR200CHG increases the IC_50_ of MDA-MB-231 cells from 2.78 ± 0.09 μg/mL to 4.62 ± 0.14 μg/mL, indicating that MIR200CHG is involved in the drug resistance process of breast cancer cells (Fig. 2n, o).

### MIR200CHG induces cancer progression

**Fig. 3:**
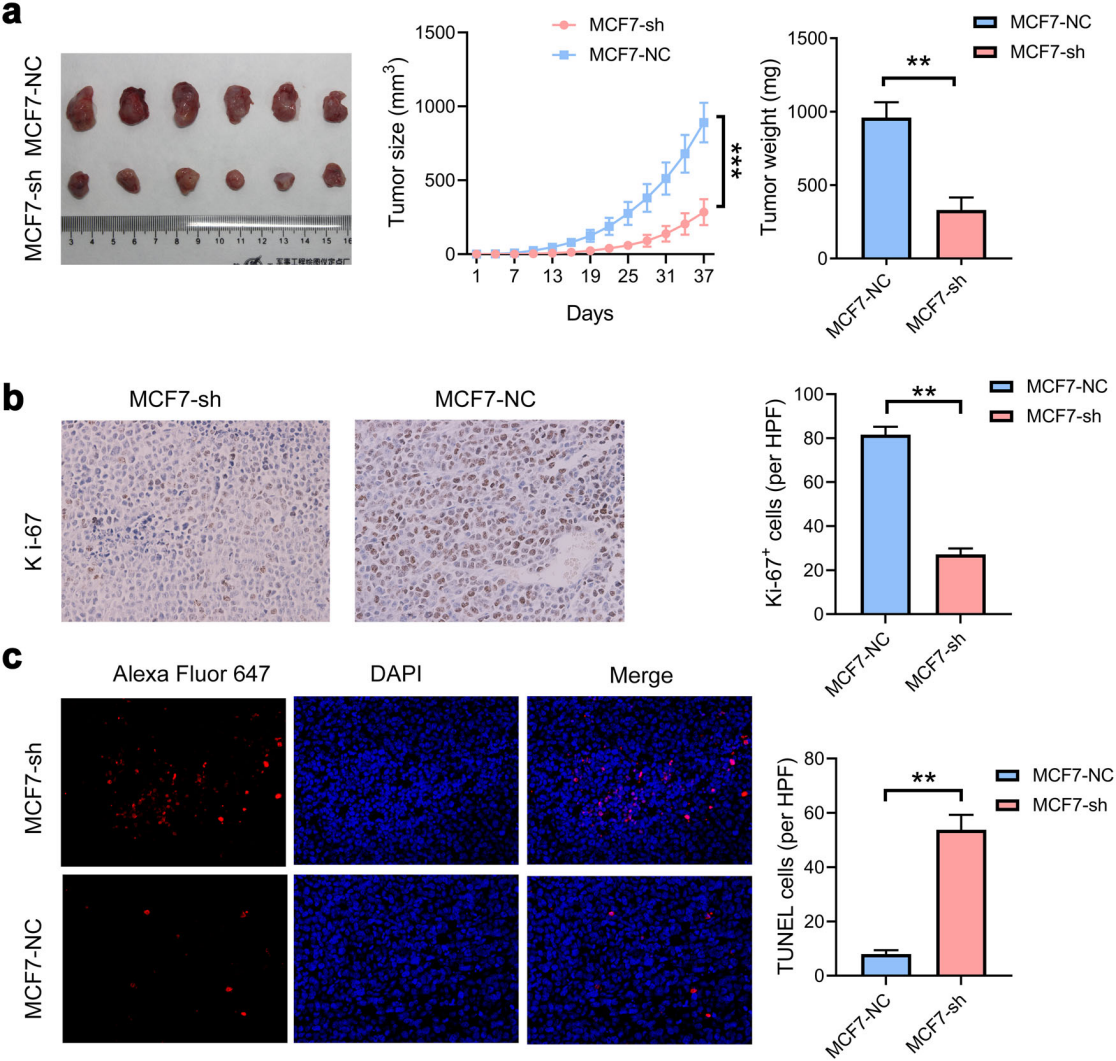
MIR200CHG promotes tumor formation in nude mice xenograft models. **a** Representative images of tumors from the MCF7-sh and MCF7-NC cell lines. The volumes and weights of the subcutaneous xenograft tumors (*n* = 6) are indicated. Tumor volumes were calculated after injection every 3 days. Data are represented as mean ± SD and were analyzed by two-way ANOVA. **P < 0.01, ***P < 0.001. **b**, **c** Representative staining images showing the expression levels of Ki-67 and TUNEL in the MCF7-sh and MCF7-NC groups (×400). Data show the mean ± SD of TUNEL and Ki-67 positive cells in five random high-power fields (HPF), and were analyzed by two-tailed *T* test. **P < 0.01.

We use nude mouse subcutaneous xenograft models to explore the effect of MIR200CHG knockdown on tumor growth in vivo. Compared with the negative control group, MCF7-sh cell tumor xenografts are smaller in size and weight (Fig. 3a). Ki-67 expression is significantly reduced in tumor grafts that knock down MIR200CHG expression (Fig. 3b). TUNEL tests demonstrate that apoptotic cells are significantly increased in tumor grafts that knock down MIR200CHG expression (Fig. 3c). Therefore, down-regulating MIR200CHG expression may be an effective way to inhibit breast cancer progression.

### MIR200CHG directly binds to YB-1

**Fig. 4:**
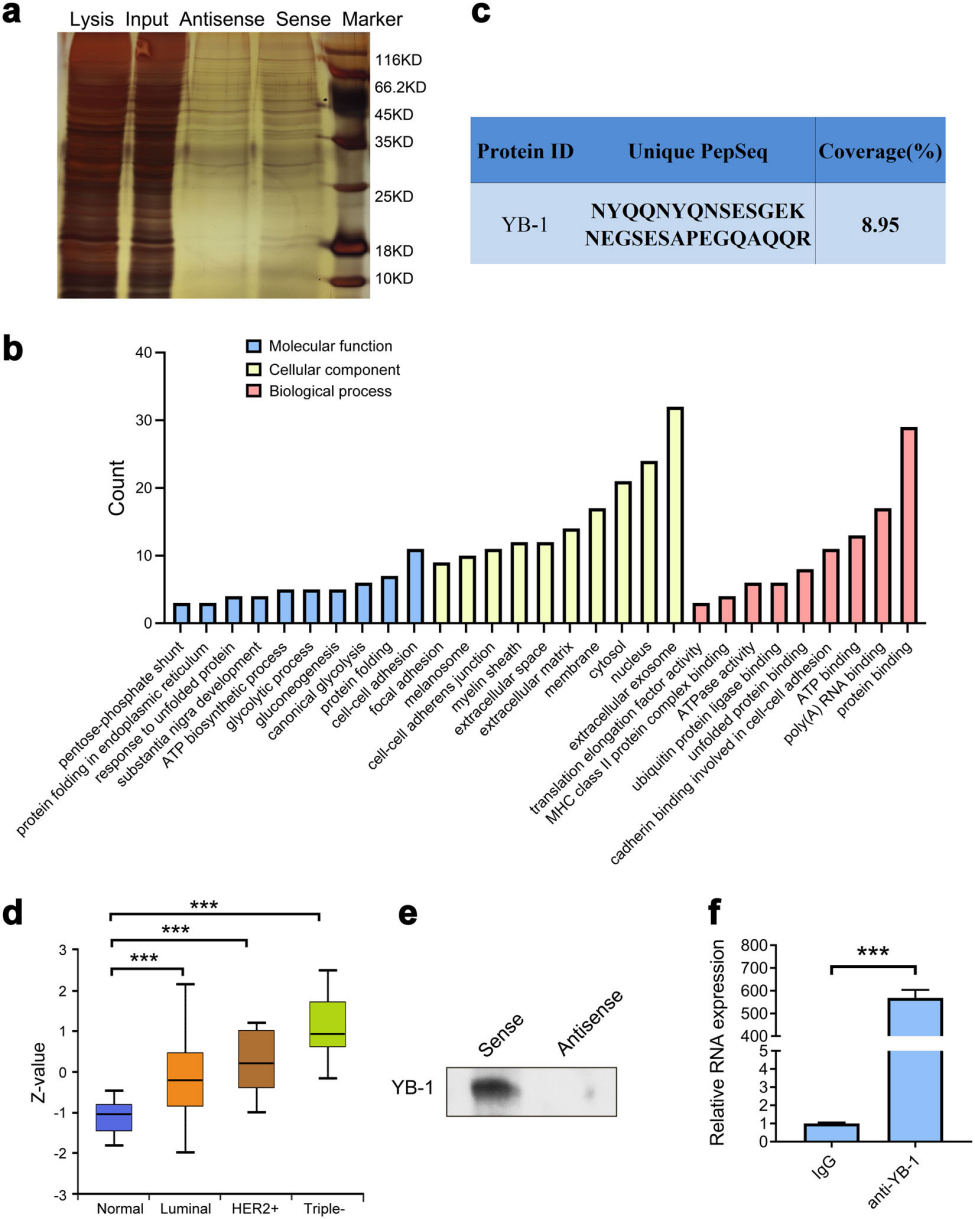
MIR200CHG directly binds to YB-1. **a** SDS-PAGE of the sense and antisense MIR200CHG pull-down protein solutions. **b** Gene ontology analysis of MIR200CHG pull-down proteins. **c** The information on the binding of MIR200CHG and YB-1 provided by mass spectrometry detection. **d** UALCAN was used to analyze the expression of YB-1 in the CPTAC breast cancer array, ***P < 0.001. Boxplots represent the median (center line) and interquartile range (IQR; box), and the whiskers represent the maximum and minimum values from the edge of the box to within 1.5 times the IQR. **e** Western blotting to detect YB-1 in sense and antisense MIR200CHG pull-down protein solutions. **f** The RIP product was isolated and purified, and the amount of MIR200CHG bound to YB-1 or IgG was measured by qPCR. IgG was used as a negative control. qPCR was performed in triplicate, and the entire RIP experiment was repeated three times. Data are shown as mean ± SEM, and analyzed by two-tailed *T* test. ***P < 0.001.

RNA pull-down and mass spectrometry are used to explore proteins that might interact with MIR200CHG. We amplify the sense and antisense MIR200CHG in vitro (Supplementary Fig. 3). After incubating the biotin-labeled sense and antisense MIR200CHG with the MCF7 cell lysate protein solution, the RNA-bound protein mixture is separated with streptavidin magnetic beads and subjected to SDS-PAGE (Fig. 4a). The pull-down protein solutions of sense and antisense MIR200CHG are analyzed by mass spectrometry, and the data are processed by Proteinpilot software. Using stringent parameters including a confidence interval of ≥95% and unique peptides ≥1, we obtain 33 proteins that bind to the sense MIR200CHG. Gene ontology (GO) analysis reveal that these proteins are widely distributed in the cytoplasm, nucleus, and exosomes, and are involved in important tumor-related processes such as intercellular adhesion and energy metabolism (Fig. 4b). Among them, the oncogenic transcription factor Y-box binding protein 1 (YB-1) arouses our interest (Fig. 4c). We use UALCAN (http://ualcan.path.uab.edu/index.html) to analyze Clinical Proteomic Tumor Analysis Consortium (CPTAC) data and find that YB-1 is upregulated in various molecular subtypes of breast cancer (Fig. 4d). We use YB-1 antibody to perform western blotting on these two pull-down protein solutions, and detect YB-1 in the protein solution pulled down by sense MIR200CHG (Fig. 4e). In addition, RIP experiments are used to verify the combination of MIR200CHG and YB-1. As expected, we detect sense MIR200CHG in the YB-1 protein precipitate of MCF7 cells (Fig. 4f).

### MIR200CHG inhibits YB-1 ubiquitination and degradation

**Fig. 5:**
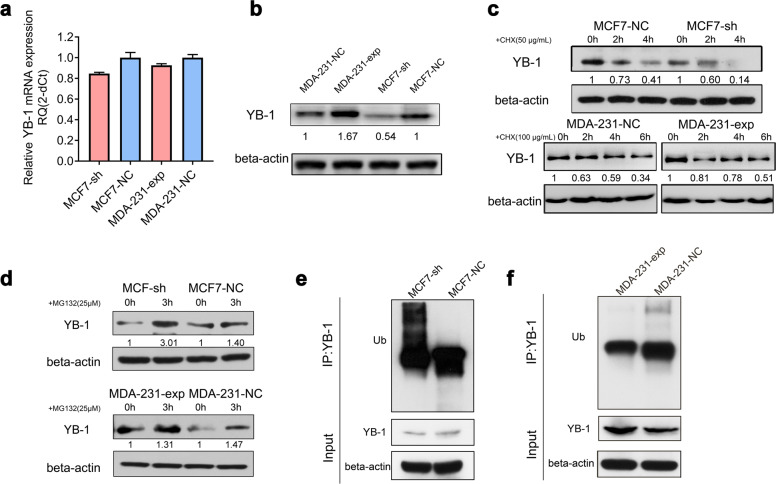
MIR200CHG inhibits YB-1 ubiquitination and degradation. **a** qRT-PCR was used to detect the changes in the *YB-1* mRNA when MIR200CHG was knocked down or overexpressed. **b** Western blotting was used to detect YB-1 expression when MIR200CHG was knocked down or overexpressed. **c** Western blotting was used to measure the expression of YB-1 in breast cancer cells following treatment with cycloheximide (CHX) after knockdown or overexpression of MIR200CHG. MCF7 cells were treated with 50 μg/mL CHX for 2 or 4 h. MDA-MB-231 cells were treated with 100 μg/mL CHX for 2, 4, or 6 h. **d** Western blotting was used to measure the expression of YB-1 in breast cancer cells that were treated with MG132 after knockdown or overexpression of MIR200CHG. Cells were treated with MG132 (25 μM) for 3 h. **e**, **f** Cell lysates of MCF7-sh and its negative control, or MDA-231-exp and its negative control were subjected to YB-1 immunoprecipitation, and then anti-Ub antibody was used to detect the levels of ubiquitinated YB-1 by western blotting. The cells were treated with MG132 (10 μM) for 24 h. Data in (**b**–**f**) are representative of at least two independent experiments.

We find that knockdown or overexpression of MIR200CHG affects the corresponding decrease or increase in YB-1 expression but does not affect *YB-1* mRNA expression (Fig. 5a, b). We treat MCF7-sh, MCF7-NC, MDA-231-exp, and MDA-231-NC cells with the protein synthesis inhibitor cycloheximide (CHX) to block de novo protein synthesis, and find that downregulation of MIR200CHG significantly accelerates YB-1 degradation, while upregulation of MIR200CHG slows down YB-1 degradation, indicating that MIR200CHG inhibits the degradation of YB-1 (Fig. 5c). We subsequently treat the two groups of cells with the proteasome inhibitor MG132, and find that YB-1 expression is partially rescued after adding MG132, which indicates that MIR200CHG is involved in the proteasome degradation pathway of YB-1 (Fig. 5d). We immunoprecipitate YB-1 in MCF7-sh, MCF7-NC, MDA-231-exp, and MDA-231-NC cells using specific antibodies and perform western blotting with ubiquitinated antibodies. Downregulation of MIR200CHG promotes YB-1 ubiquitination, while overexpression of MIR200CHG inhabits YB-1 ubiquitination (Fig. 5e, f). Therefore, MIR200CHG can regulate YB-1 expression at the post-transcriptional level by inhibiting the ubiquitin-proteasome pathway.

### MIR200CHG promotes YB-1 phosphorylation at serine 102

**Fig. 6:**
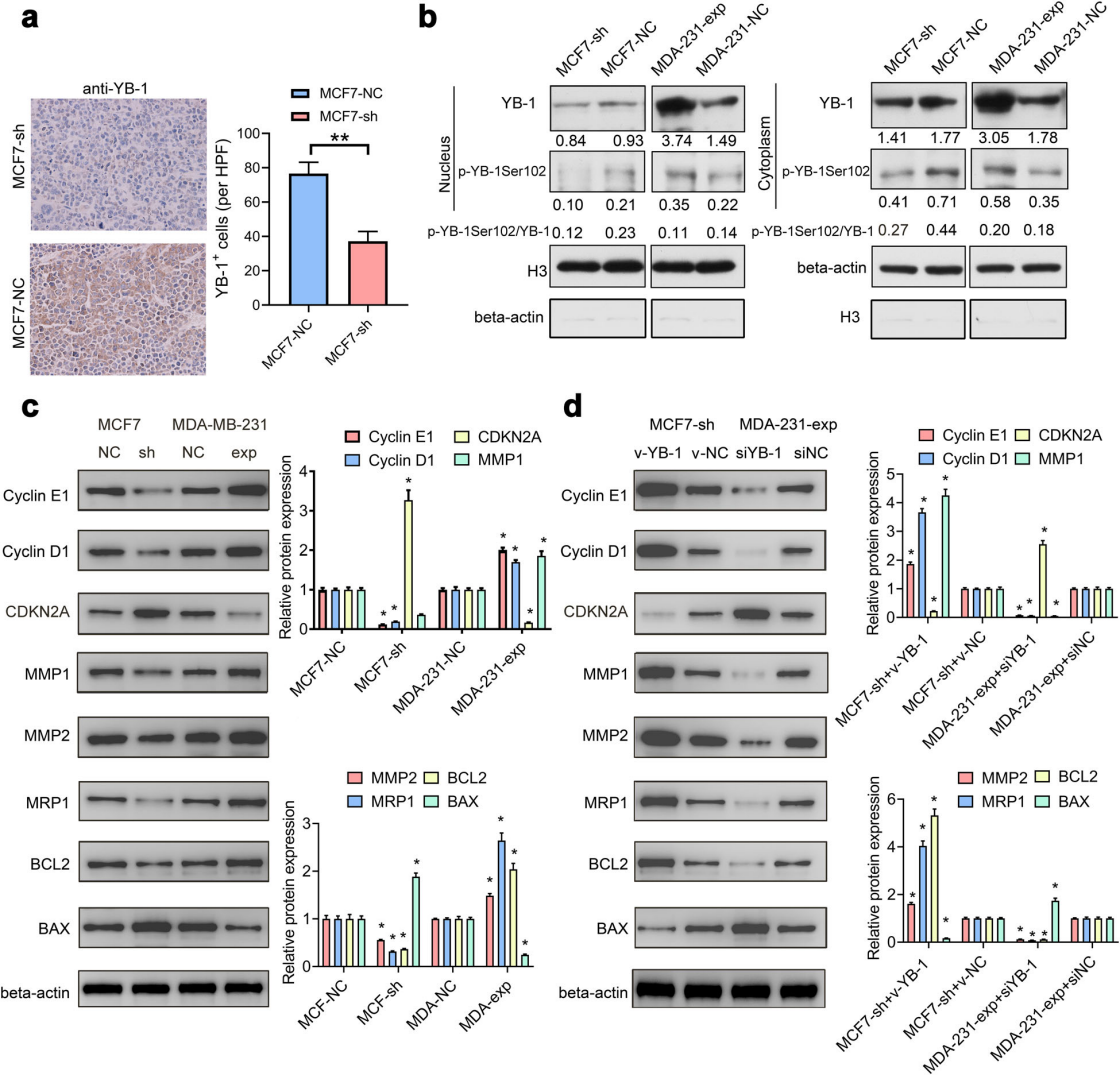
MIR200CHG promotes YB-1 phosphorylation at serine 102 and nuclear translocation. **a** Representative images of immunohistochemical staining showing the expression level of YB-1 in the MCF7-sh and MCF7-NC cell line groups (400×). Data show the mean ± SD of YB-1 positive cells in five random high-power fields (HPF), and were analyzed by two-tailed *T* test. **P < 0.01. **b** Western blot was used to detect the impact of MIR200CHG knockdown and overexpression on the expression of p-YB-1Ser102 and YB-1 in the nucleus or cytoplasm of MCF7 and MDA-MB-231 cells. Histone H3 was used as an internal reference for nuclear proteins, and β-actin was used as an internal reference for cytoplasmic proteins. Data are representative of at least two independent experiments. **c** Western blot was used to detect the changes associated with the knockdown or overexpression of MIR200CHG in the YB-1 related proteins cyclin D1, cyclin E1, CDKN2A, MMP1, MMP2, MRP1, BCL2, and BAX in MCF7, and MDA-MB-231 cells. **d** Western blot was used to detect the effects of YB-1 interference or overexpression on the related proteins cyclin D1, cyclin E1, CDKN2A, MMP1, MMP2, MRP1, BCL2, and BAX in MCF7-sh and MDA-231-exp cells. Data in (**c**, **d**) were confirmed in three independent experiments, represented as mean ± SD, and analyzed by two-tailed *T* test. *P < 0.05.

We perform YB-1 immunohistochemical staining using tumor tissues in the xenograft models, and find that YB-1 is significantly reduced in the tumor cells with downregulated MIR200CHG expression (Fig. 6a). Subsequently, we extract the nuclear and cytoplasmic proteins of MCF7-sh, MCF7-NC, MDA-231-exp, and MDA-231-NC for western blotting. The results show that downregulation of MIR200CHG reduces p-YB-1Ser102 expression in the nucleus and cytoplasm, and is accompanied by a decrease in the ratio of p-YB-1Ser102/YB-1, while upregulation of MIR200CHG increases p-YB-1Ser102 expression in the nucleus and cytoplasm, although the ratio of p-YB-1Ser102/YB-1 does not change significantly (Fig. 6b). We also test several proteins known to be regulated by YB-1 nuclear translocation, which play an important role in cell proliferation, apoptosis, and drug resistance: cyclin D1, cyclin E1, CDKN2A, MMP1, MMP2, MRP1, BCL2, and BAX. All these proteins demonstrate responsive changes to MIR200CHG. In MCF7-sh cells, cyclin D1, cyclin E1, MMP1, MMP2, MRP1, and BCL2 are downregulated, while CDKN2A and BAX are upregulated. In MDA-231-exp cells, cyclinD1, cyclin E1, MRP1, MMP1, MMP2, and BCL2 are upregulated, while CDKN2A and BAX are downregulated (Fig. 6c). Moreover, we perform rescue experiments: transfect *YB-1* expression vector (v-YB-1) and negative control (v-NC) in MCF7-sh cells, and transfect *YB-1* interference fragment (siYB-1) and negative control (siNC) in MDA-231-exp cells. Accordingly, when the changes in the expression of YB-1 in cells are “rescued”, the expressions of CDKN2A, cyclin D1, cyclin E1, MMP1, MMP2, MRP1, BCL2, and BAX all demonstrate corresponding changes (Fig. 6d).

### MIR200CHG partially affects miR-200c/141-3p expression

**Fig. 7:**
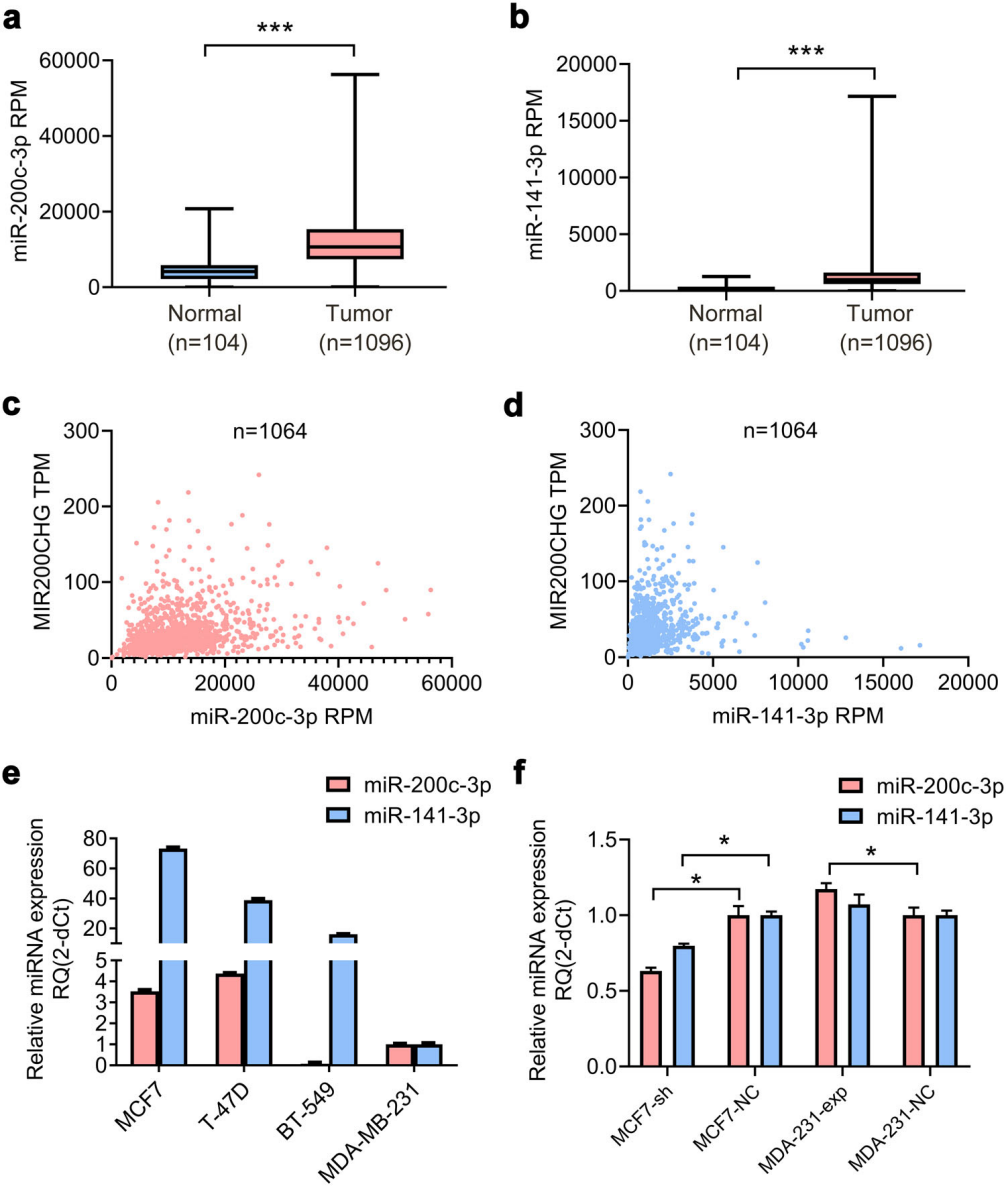
Correlation between the expression of MIR200CHG and miR-200c/141-3p. **a**, **b** Analysis of the expression of miR-200c/141-3p in the TCGA breast cancer array. ***P < 0.001. Boxplots represent medians (center line) and interquartile range (IQR; box), and whiskers represent the maximum and minimum values within 1.5 times the IQR from the edge of the box. **c**, **d** Correlations between the expression of MIR200CHG and miR-200c/141-3p were analyzed by TCGA breast cancer array. **e** Expression of miR-200c/141-3p in breast cancer cell lines (MCF7, T-47D, BT-549, MDA-MB-231) was analyzed by qRT-PCR. Data were confirmed in three independent experiments and are represented as mean ± SEM in triplicates. **f** The effect of MIR200CHG knockdown or overexpression on the expression of miR-200c/141-3p in breast cancer cells was analyzed by qRT-PCR. The experiment was carried out in triplicate and repeated at least three times. Data are represented as mean ± SEM, and analyzed by two-tailed *T* test. *P < 0.05.

MiR-200c and miR-141 are located in the intron region of the *MIR200CHG* gene, and miR-200c/141-3p processed from the 3′ end arm is the focus of functional research. miRBase (http://www.mirbase.org/) and ENCORI (http://starbase.sysu.edu.cn/) indicate that MIR200CHG is not a target of miR-200c/141-3p. The miRNA-Seq array in the TCGA shows that miR-200c/141-3p is highly expressed in breast cancer tissues compared to normal breast (Fig. 7a, b). Pearson correlation analysis reveals that miR-200c/141-3p is weakly correlated with the expression of MIR200CHG (miR-200c-3p, *r* = 0.2888; miR-141-3p, *r* = 0.1595) (Fig. 7c, d). The expression trends of miR-200c/141-3p and MIR200CHG are not completely synchronized in breast cancer cells (Fig. 7e). Additionally, downregulation MIR200CHG reduces miR-200c-3p to 63.3% and miR-141-3p to 79.8% in MCF7 cells, while upregulating MIR200CHG increases miR-200c-3p to 117.3% in MDA-MB-231 cells. However, miR-141-3p does not change significantly (Fig. 7f).

## Discussion

LncRNAs are key regulators of gene expression and remodeling in eukaryotic genomes. They are dysregulated in several cancers and increasingly represent cancer treatment targets^15^. We find that MIR200CHG is significantly upregulated in breast cancer and is related to the tumor size and histopathological grade. In vivo and in vitro functional experiments we show that MIR200CHG enhances breast cancer proliferation, invasion, and drug resistance. Moreover, MIR200CHG can activate the transcription of multiple tumor-related proteins by regulating the ubiquitination and phosphorylation status of YB-1, thereby promoting the progression of breast cancer.

We find that MIR200CHG and YB-1 bind stably in breast cancer cells, and the changes in the expression of MIR200CHG do not affect the level of *YB-1* mRNA but affect the post translational modification status of YB-1. The immunohistochemical results of tumor tissues in the xenograft models confirm the effect of MIR200CHG on YB-1 expression: YB-1 belongs to the evolutionary conserved Y-box protein family and is a multifunctional protein that can bind single-stranded DNA and RNA^16^. YB-1 is involved in basic gene expression processes, including transcription, translation, and mRNA stabilization^17^. Studies have confirmed that YB-1 can interact with E3 ubiquitin ligase through the highly conserved cold shock protein domain (CSD) and can undergo polyubiquitination and degradation through the proteasome pathway^18,19^. The ubiquitin-proteasome system is responsible for the degradation of cytoplasmic, nuclear, and endoplasmic reticulum proteins in eukaryotic cells except lysosome^20^. There have been studies on lncRNA inhibiting YB-1 degradation through the ubiquitin-proteasome pathway to regulate its function. For example, the lncRNA LNCAROD acts as a scaffold for the interaction between YB-1 and HSPA1A, prevents YB-1 proteasome degradation in head and neck squamous cell carcinoma (HNSCC) cells, and promotes the malignant behavior of HNSCC cells^21^. LncRNA PIK3CD-AS2 binds to and maintains the stability of YB-1, inhibits p53 signal transduction by protecting YB-1 protein from ubiquitination degradation, thereby promoting the progression of lung adenocarcinoma^22^. Our results show that knockdown or overexpression of MIR200CHG does not affect the expression of *YB-1* mRNA, but can inhibit the degradation of YB-1 by inhibiting the ubiquitin-proteasome pathway. Since MIR200CHG is more highly expressed in luminal A breast cancer and YB-1 is more highly expressed in triple-negative breast cancer, we believe that the endogenous expression of YB-1 in breast cancer is regulated by more other factors.

In addition to participating in YB-1 ubiquitination, we find that MIR200CHG can also positively regulate the phosphorylation of serine 102 on the cold shock protein domain of YB-1, thereby promoting YB-1 nuclear translocation events. YB-1 is mainly located in the cytoplasm and partly in the nucleus^23^. YB-1 in the cytoplasm is the main component of the mRNA-protein complex (mRNP), which is related to the stability of the RNA transcript and regulates the mRNA translation process in a concentration-dependent manner^24^. When cells undergo environmental stimulation, YB-1 is affected by the PI3K/Akt and MAPK/ERK signaling pathways, and the serine residue 102 on its CSD is phosphorylated, which promotes nuclear translocation^25,26^. Ser102 phosphorylation not only helps YB-1 as a transcription factor to activate or inhibit the expression of related genes, but also helps YB-1 to participate in the DNA repair process^27^. Our study confirms that MIR200CHG is involved in the phosphorylation regulation of YB-1 serine 102, but it is not clear how MIR200CHG activates YB-1 phosphorylation. MIR200CHG may act as a scaffold for an extracellular signal-regulated kinase and YB-1 to regulate phosphorylation, or MIR200CHG may bind to YB-1 and block the dephosphorylation of phosphatase. More research is needed to elucidate the mechanism.

We have demonstrated that MIR200CHG can inhibit the degradation of YB-1 protein by inhibiting the ubiquitin-proteasome pathway, and can regulate the phosphorylation state of YB-1 to affect its intracellular distribution. The accumulation of YB-1 in breast cancer cell nuclei is considered to be an independent prognostic factor for overall and progression-free survival^28,29^. YB-1 inhibits CDKN2A transcription and activates cyclin D1, and the expression of cyclin E1 promotes the cell cycle process^30,31^. YB-1 is considered to be a negative regulator of p53, which hinders the transcription of p53-dependent pro-apoptotic genes, and can also act on the BCL2 protein family to inhibit the mitochondrial apoptosis pathway^32,33^. Matrix metalloproteinases are a group of endopeptidases or proteolytic enzymes, which are involved in the migration and invasion of cancer cells, angiogenesis, tissue remodeling, and differentiation^34^. YB-1 can act as a transcription factor or transcription activator to promote the expression of MMP1/MMP2^35–37^. YB-1 also activates the transcription of MRP1 to enhance the resistance of tumor cells to cisplatin, 5-fluorouracil, paclitaxel, and doxorubicin, thereby promoting tumor cell resistance^25,38^. Through gain and loss of function and rescue experiments, we confirm that MIR200CHG regulates the expression of tumor-associated proteins CDKN2A, cyclin D1, cyclin E1, BCL2, BAX, MMP1, MMP2, and MRP1 by binding YB-1, thereby promoting breast cancer proliferation, invasion, and resistance.

In addition to co-transcription with host genes, intronic miRNAs are most often directly transcribed independently of the host genes^10^. Studies have confirmed that miR-200c/141 have their own independent transcription initiation sites^39,40^. We analyze the TCGA data and find that MIR200CHG and miR-200c/141-3p are weakly correlated in expression, while the expression of miR-200c-3p is more correlated with MIR200CHG. The detection results in breast cancer cell lines show that the expression of MIR200CHG in MDA-MB-231 cells is the lowest, which may be related to the DNA methylation of the MIR200CHG promoter region in MDA-MB-231 cells^41,42^. Knockdown of MIR200CHG partially affects the expression of miR-200c /141-3p, which indicates that there may be functional crosstalk between MIR200CHG and miR-200c/141-3p. The most direct functional association between lncRNA and miRNA is to act as miRNA sponges^43^. Our analysis shows that MIR200CHG cannot be used as a competitive endogenous RNA for miR-200c/141. Furthermore, host and intronic miRNAs can also function to produce crosstalk by regulating the common molecular target^44^. Although there is no report on the function of YB-1 and miR-200c/141, we search ENCORI^45^, and find that there are multiple miR-200c/141 target genes in the mass spectrometry results of the MIR200CHG pull-down protein solution, indicating that MIR200CHG and miR-200c/141 may be functionally related.

In summary, we confirm that MIR200CHG is upregulated in breast cancer and is related to the tumor size and histopathological grade. MIR200CHG affects the ubiquitination and phosphorylation at serine 102 of YB-1 to regulate the expression of genes related to tumor cell proliferation, invasion, and drug resistance. Targeting MIR200CHG may help to overcome the progression and chemotherapy resistance of breast cancer, and provide a new strategy for using lncRNA as a molecular target for breast cancer treatment.

## Methods

### Chemicals and reagents

Leibovitz’s L-15 medium, trypsin-EDTA (0.25%), and fetal bovine serum (FBS) were procured from Gibco (Grand Island, NY, USA). MycoProb Mycoplasma Detection Kit was purchased from R&D Systems (Minneapolis, MN, USA). cOmplete EDTA-free Protease Inhibitor Cocktail and PhosSTOP phosphatase inhibitor Cocktail were purchased from Sigma-Aldrich (St. Louis, MO, USA). The antibodies used in this study were purchased from Abcam (Cambridge, MA, USA) and Cell Signaling Technology (Danvers, MA, USA), and the relevant information is provided in Supplementary Note 1. Fluorescence in situ hybridization (FISH) kit was purchased from GenePharma (Shanghai, China). Puromycin dihydrochloride, PARIS Kit, Lipofectamine 3000, and TRIzol reagent were purchased from Invitrogen (Carlsbad, CA, USA). Cisplatin was purchased from Qilu Pharmaceutical Co., Ltd. (Jinan, China). Cell Counting Kit-8 (CCK-8) was purchased from Dojindo Laboratories (Kyushu Island, Japan). Standard Bradford Assay Kit, Cell Cycle Detection Kit, and Annexin V-APC/7-AAD Apoptosis Detection Kit were purchased from KeyGEN (Nanjing, China). Pierce Magnetic RNA-Protein Pull-Down Kit and SuperSignal West Femto Maximum Sensitivity Substrate Kit were purchased from Thermo Scientific (Waltham, MA, USA). Magna RIP RNA-Binding Protein Immunoprecipitation Kit and PureProteome Protein A/G Mix Magnetic Beads were purchased from Merck Millipore (Billerica, MA, USA). BioCoat (Matrigel matrix) Tumor Invasion Systems was purchased from Corning (Corning, NY, USA).

### Clinical tissue specimens and cell lines

Breast cancer tissues and adjacent normal tissues were collected in the Affiliated Cancer Hospital of Nanjing Medical University from women who had undergone breast cancer surgery from 2018 to 2019, and were immediately stored in liquid nitrogen until RNA extraction. At least two pathologists confirmed the histopathological diagnoses of these samples. This study was approved by the Affiliated Cancer Hospital Research Committee of Nanjing Medical University. All subjects gave informed consent in accordance with the Declaration of Helsinki. Breast cancer cell lines MCF7, T-47D, BT549, and MDA-MB-231 were obtained from the Cell Bank of the Chinese Academy of Sciences (Shanghai, China), cultured according to the ATCC protocol, and regularly tested for mycoplasma.

### RNA extraction and qRT-PCR

Total RNA was isolated from cells and tissues using TRIzol reagent. Nuclear and cytoplasmic components were separated using the PARIS Kit according to the manufacturer’s instructions. Polymerase chain reaction (PCR) amplification was carried out by a two-step method, the cycle parameters were: pre-denaturation at 95 °C for 30 s, then 40 cycles of: 95 °C for 5 s, 56 °C for 20 s, and finally melting curve analysis. The entire PCR reaction and analysis were performed in the LightCycler 2.0 PCR system (Roche, Basel, Switzerland). We analyzed the fluorescence signal in the amplification and melting curves, and the average cycle threshold (Ct) values were calculated, followed by calculation of the relative expressions of the target genes by the 2^−ΔΔCt^ method. The relative gene expressions of miR-200c-3p and miR-141-3p were calculated with U6. Bulge-loop^TM^ miRNA qRT-PCR Primer Sets specific for miR-200c-3p, miR-141-3p, and U6 were purchased from Ribobio (Guangzhou, China). Relative gene expressions of MIR200CHG and *YB-1* mRNA were calculated with GAPDH. The primer sequences of MIR200CHG, YB-1, and GAPDH are listed in Supplementary Note 2.

### Interference and overexpression experiments

MIR200CHG shRNA lentiviral particles were synthesized and packaged by GeneChem (Shanghai, China) (Supplementary Note 3), and used to infect MCF7 cells with multiplicity of infection (MOI) = 20. After 72 h of infection, puromycin (5 μg/mL) was added to screen the MCF7 cells that stably expressed the MIR200CHG shRNA fragment (MCF7-sh) and the negative control (MCF7-NC). MIR200CHG lentiviral overexpression particles were synthesized and packaged by GenePharma (Supplementary Note 4), and used to infect MDA-MB-231 cells with MOI = 15. After 72 h of infection, puromycin (2 μg/mL) was added to screen the MDA-MB-231 cells stably overexpressing the MIR200CHG fragment (MDA-231-exp) and the negative control (MDA-231-NC). Small interfering RNA siYB-1 and negative control (siNC) were purchased from GenePharma. The cDNA sequence of *YB-1* was amplified in vitro and cloned into the *BamH* I and *Nhe* I sites of the pcDNA3.1 vector, and labeled with the negative control as v-YB-1 and v-NC, respectively (Supplementary Note 5). Both small interfering RNA and plasmid DNA were transfected with Lipofectamine 3000.

### Xenograft model

Xenograft models were used to evaluate the effect of MIR200CHG knockdown on tumor growth in vivo. MIR200CHG knockdown cell line MCF7-sh and its negative control MCF7-NC were resuspended in 0.1 mL potassium buffered saline (PBS) (>2 × 10^6^ cells) and injected into the fat pads of 5–6 weeks old female BALB/c nude mice. Subsequently, we used a digital caliper to measure the length (mm) and width (mm) of the tumor, calculated the tumor volume (mm^3^) according to the following formula: (length × width^2^)/2, and drew a growth curve. The mice were sacrificed after 37 days, and the tumor tissues were excised and weighed. Tumor tissue paraffin sections were prepared and used for Ki-67, YB-1 immunohistochemical staining, and TUNEL immunofluorescence detection. All animal studies were approved by the Institutional Animal Care and Use Committee of Hubei Medical College of Preventive Sciences and conducted in accordance with the National Institutes of Health’s Guide for the Care and Use of Laboratory Animals.

### FISH experiment

The MIR200CHG probes were labeled with CY3, and the probe sequences are listed in Supplementary Note 6. We placed the cell slides in a 24-well plate, seeded the cells at a density of 1 × 10^4^ cells per well, and incubated overnight at 37 °C. After the medium in each well was discarded and it was washed twice with PBS, 1 ml of 4% paraformaldehyde was added and fixed at room temperature for 15 min. After blocking, the cells were incubated with the labeled probes for 16 h and washed three times with a washing solution in an oven at 60 °C for 30 min each time. Subsequently, we added DAPI fluorescent dye solution for 20 min at room temperature while avoiding light, and then observed the distribution of MIR200CHG in the cells under a confocal microscope.

### RNA pull-down and mass spectrometry analysis

The sense and antisense MIR200CHG were amplified in vitro, and labeled with desulfurization biotinylation, followed by incubation with streptavidin magnetic beads. All experiments were performed in accordance with the manufacturer’s instructions, and the proteins that interacted with the sense or antisense MIR200CHG were separated. The proteins were detected by Triple TOF 5600-plus instrument (AB Sciex, Redwood City, California, USA) and analyzed by Proteinpilot software. The primers for sense MIR200CHG and antisense MIR200CHG are listed in Supplementary Note 2.

### RIP-qPCR

The RNA immunoprecipitation (RIP) was performed strictly in accordance with the instructions. Each RIP reaction required 100 μL of 2 × 10^7^ MCF7 cell lysate, and each immunoprecipitation required 5 μg of antibody. The expression of MIR200CHG in the precipitates of anti-YB-1 and negative control (IgG) was determined by quantitative reverse transcription (qRT)-PCR, and the content in the IgG precipitate was used as a reference. qRT-PCR was performed as described above.

### Immunoprecipitation and ubiquitination analysis

We seeded MCF7-sh, MCF7-NC, MDA-231-exp, and MDA-231-NC cells into 10 cm plates, added the proteasome inhibitor MG132 (10 μM), and incubated for 24 h. The cells were collected and washed three times with pre-cooled PBS, lysed with RIPA, and 10 μL of the cell lysate was taken as the input. We mixed the total cell lysate (100 μL/tube), protease inhibitor, anti-YB-1 (2 μg/tube), and PureProteome Protein A/G Mix Magnetic Beads, and incubated the tube overnight at 4 °C with rotation. Subsequently, the tube was put on a magnetic stand and the supernatant discarded, followed by washing the magnetic beads thoroughly with the washing buffer. Then, 60 μL of 1× SDS-PAGE loading buffer was added, boiled for 5 min, and finally the proteins were detected by western blot.

### FACS analysis

For cell cycle analysis, propidium iodide (PI) staining method was used. Cells from the experimental and control groups were collected separately, ensuring that the cell number in each test sample was above 10^6^. Then the cells were washed three times with cold PBS and fixed with 70% ethanol overnight at −20 °C. The fixative was washed off with PBS, followed by centrifugation at 1000 rpm for 3 min. The supernatant was discarded, 500 μL PI/RNase A staining working solution was added, and reacted for 30–60 min in the dark at room temperature. For apoptosis analysis, Annexin-V APC/7-AAD double staining method was used. Cells from the experimental and control groups were collected separately, ensuring that the number of cells in each test sample was above 10^6^. Cells were washed three times with PBS, centrifuged at 1000 rpm for 3 min, and the supernatant discarded. Then, we added 500 μL Binding Buffer, 5 μL Annexin V-APC, and 5 μL 7-AAD to resuspend the cells, and reacted for 5–15 min at room temperature in the dark. Immediately after the staining, the cell cycle and apoptosis percentage were analyzed by FACSCalibur flow cytometer (FACSCalibur, BD Biosciences, San Jose, CA, USA) and CellQuest software.

### Cell proliferation experiment

The cells were seeded in 96-well culture plates (3 × 10^3^ cells per well). After culturing for 0, 24 h, 48 h, 72 h, and 96 h, culture solution in each well was replaced with 100 μL of a 10% CCK-8 reaction solution. After incubation at 37 °C for 4 h, the absorbance was measured at 450 nm. For drug sensitivity testing, cells were seeded in 96-well plates at 5 × 10^3^ cells per well. They were treated with different concentrations of cisplatin for 48 h, and then the medium in each well was replaced with 100 μL of 10% CCK-8 reaction solution. The wells treated with the same volume of cisplatin solvent (physiological saline) were added as negative control groups. After incubation at 37 °C for 4 h, the absorbance was measured at 450 nm, and the half maximal inhibitory concentration (IC_50_) value of each group was calculated.

### Colony formation experiment

The plate colony formation experiment was used to test the in vitro proliferation ability of single cells. We prepared single cell suspensions of MCF7-sh, MCF7-NC, MDA-231-exp, and MDA-231-NC, seeded them in 6-well plates (800 cells per well), and placed them in a 37 °C incubator. When visible colonies appeared in the culture plates, they were fixed with 4% paraformaldehyde and stained with 0.1% crystal violet staining solution.

### Wound healing experiment

The cells were seeded in 6-well plates at a density of 5 × 10^5^ cells per well and incubated with cell culture medium at 37 °C overnight. An artificial wound was made with a sterile tip, and the fragments were washed thoroughly with PBS. We use a microscope to detect cell migration near the wound and obtain images. MCF7 cells were incubated for 20 h, and MDA-MB-231 cells were incubated for 6 h. The images were processed using Image J software to quantify the open wound area as average open wound area % and a histogram was drawn.

### Cell invasion experiment

The cells were harvested, resuspended in 1% serum medium, and seeded in Matrigel matrix-plated chambers (2 × 10^4^ cells per well). After incubating for different durations according to the differences in the cell invasion ability, we manually removed the cells that had not migrated through the Matrigel matrix and the small holes with cotton swabs. The chambers were fixed with 4% formaldehyde, stained with 0.1% crystal violet, and five fields of view were randomly selected under the microscope to count the cell number.

### Western blotting

Protease inhibitor cocktail tablets and phosphatase inhibitor cocktail tablets were added to RIPA buffer. After the cells were sufficiently lysed, the standard Bradford method was used to measure the protein content of the lysate. The proteins were separated on 10% SDS-PAGE, and transferred to polyvinylidene fluoride membranes using a Trans-Blot SD Semi-Dry Transfer Cell (Bio-Rad, Hercules, CA, USA). After blocking with 5% BSA in TBST, the membranes were separately incubated overnight at 4 °C with antibodies for anti-beta-actin (1:2000), anti-histone H3 (1:2000), YB-1 (1:1000), p-YB-1Ser102 (1:1000), MRP1 (1:1000), MMP1 (1:1000), MMP2 (1:1000), CDKN2A (1:1000), cyclin D1 (1:1000), cyclin E1 (1:1000), BCL2 (1:1000), and BAX (1:1000). Subsequently, the membranes were washed and incubated with horseradish peroxidase conjugated secondary antibodies for 1 h. The bands were detected using a SuperSignal West Femto Maximum Sensitivity Substrate Kit, images were acquired using a SYNGENE G: BOX chemiXR5 System (Cambridge, UK), and the results were analyzed using Gel-Pro32 software. All blots were derived from the same experiment and were processed in parallel. Beta-actin was used as an internal reference and histone H3 was used as an internal reference for nuclear proteins.

### Statistical analysis

Mann–Whitney *U* test was used to analyze the clinicopathological data to detect the differences in MIR200CHG expression in breast cancer. Receiver operating characteristic (ROC) curve and area under the curve (AUC) analysis were used to estimate the trade-off between sensitivity and specificity at different possible cut-offs for diagnostic testing using MIR200CHG. Two-way ANOVA or two-tailed *T*-test was used to evaluate the significant differences in cellular experiments between the groups. Two-tailed unpaired *T* test was used to evaluate the difference in the expression of MIR200CHG in breast cancer tissues and adjacent tissues. Pearson correlation coefficient was used to analyze the correlation between MIR200CHG and miR200c/141-3p. *P* value less than 0.05 was considered statistically significant. Data were analyzed using R Project, GraphPad Prism 8 (GraphPad Software, San Diego, CA, USA), and SPSS version 19.0 (SPSS, Chicago, IL, USA).

### Reporting summary

Further information on research design is available in the Nature Research Reporting Summary linked to this article.

## Supplementary information

Supplementary Information

Reporting Summary

## Data Availability

The data generated and analyzed during this study are described in the following data record: 10.6084/m9.figshare.14731272^46^. The lncRNA microarray data of breast cancer tissues have been deposited in NCBI Gene Expression Omnibus database (GEO) and are openly available via accession https://identifiers.org/geo:GSE115275^47^. The mass spectrometry results of RNA pull-down protein solutions have been deposited in PRIDE^48^ and are available via accession https://identifiers.org/pride.project:PXD026480^49^. The TCGA data used in this study is publicly available on the National Cancer Institute’s (NCI) Genomic Data Commons (GDC) (https://portal.gdc.cancer.gov/). The search terms used to locate the data in GDC were: BRCA RNAseq: breast, TCGA-BRCA, transcriptome profiling, HTseq-FPKM; BRCA miRNAseq: breast, TCGA-BRCA, transcriptome profiling, Isoform Expression Quantification, miRNA-Seq, BCGSC miRNA Profiling. Under reasonable circumstances, the materials generated in this study can be obtained from the corresponding authors. The raw data of all Western blots are provided in Supplementary Fig. 5.

## Code availability

This study did not use custom code or scripts.
