# Supplementary material for: Long non-coding RNA MIR200CHG promotes breast cancer proliferation, invasion, and drug resistance by interacting with and stabilizing YB-1
Source: NPJ Breast Cancer. 2021 Jul 16;7:94. doi: 10.1038/s41523-021-00293-x (PMC8285504; doi:10.1038/s41523-021-00293-x)
Supplement: Supplementary file 1 — Supplementary Information [file 41523_2021_293_MOESM1_ESM.pdf]

Supplementary Figure 1. The expression of MIR200CHG was analyzed by lncRNA microarray and TCGA data.

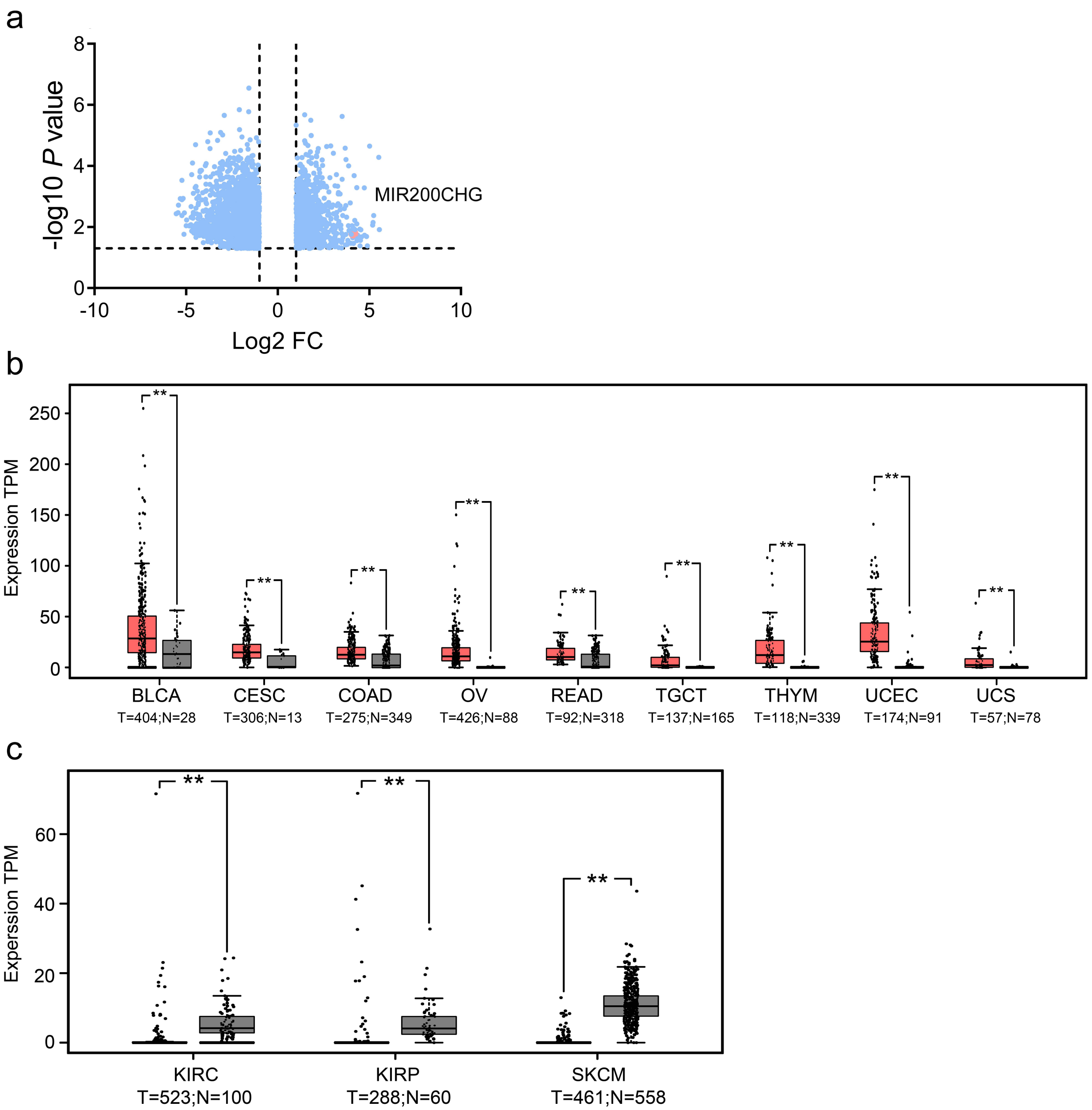

(a) Volcano plot showing differentially expressed lncRNAs (fold change ≥ 2-fold, $P < 0.05$) in lncRNA microarray (GSE115275) analysis. Red dot, MIR200CHG; fold change = 17.042; $P = 0.022$.

(b) GEPIA2 indicates that MIR200CHG is up-regulated in a variety of cancers in The Cancer Genome Atlas. Bladder urothelial carcinoma (BLCA), cervical and endocervical cancers (CESC), colon adenocarcinoma (COAD), ovarian serous cystadenocarcinoma (OV), rectum adenocarcinoma (READ), testicular germ cell tumor (TGTC), thymoma (THYM), uterine corpus endometrial carcinoma (UCEC), uterine carcinosarcoma (UCS). ** $P < 0.01$.

(c) GEPIA2 shows that MIR200CHG is down-regulated in certain cancers in The Cancer Genome Atlas. Kidney renal clear cell carcinoma (KIRP), kidney renal papillary cell carcinoma (KIRC), skin cutaneous melanoma (SKCM). N, non-tumor tissue, gray. T, tumor tissue, red. ** $P < 0.01$.

Supplementary Figure 2. MIR200CHG promotes a variety of malignant behaviors of breast cancer cells in vitro.

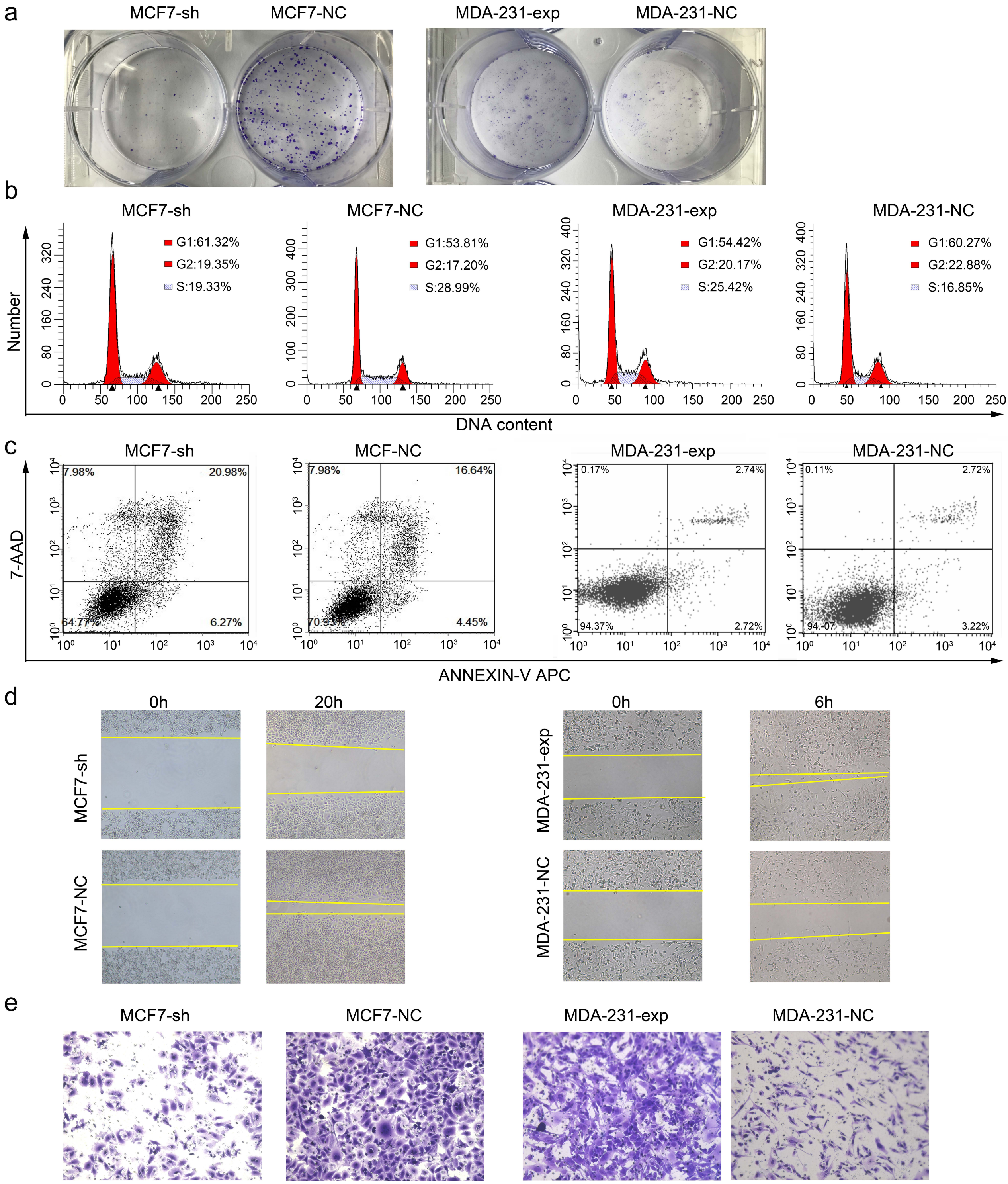

(a-e) Representative images of MCF7 and MDA-MB-231 cell colony formation experiments, cell cycle detection, apoptosis detection, wound healing experiments, and transwell cell invasion experiments when MIR200CHG was knocked down or overexpressed.

Supplementary Figure 3.

Gel electrophoresis of the sense and antisense MIR200CHG PCR transcripts.

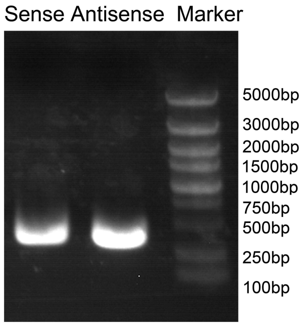

Supplementary Figure 4. Plot the detaCT values in qRT-PCR. *, $P < 0.05$. **, $P < 0.01$.

Figure 2a

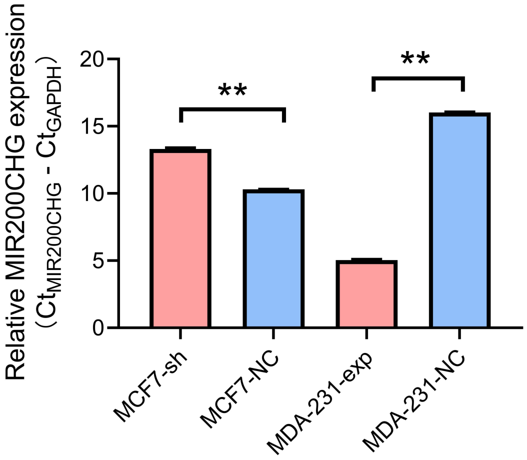

Figure 5a

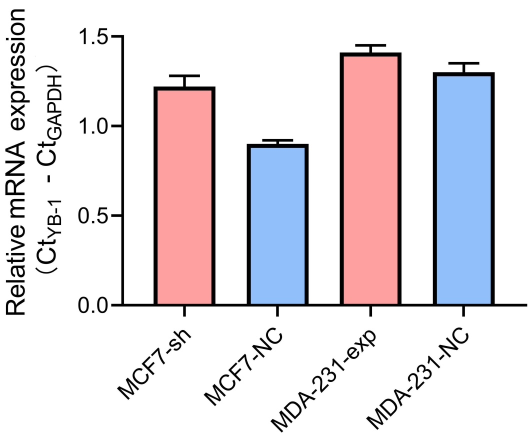

Fiigure 7e

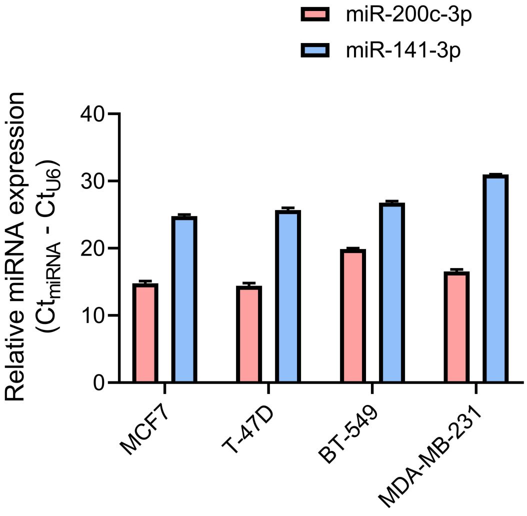

Figure 7f

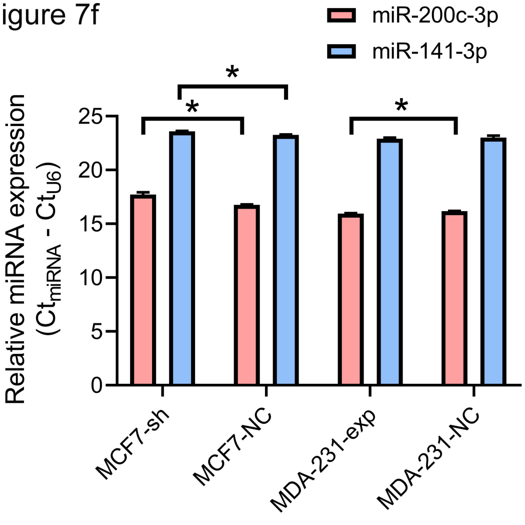

Supplementary Figure 5. Raw data for the Western blot.

Figure 4e

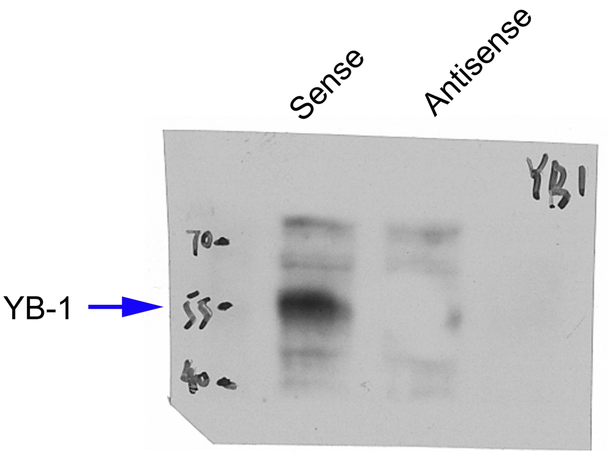

Figure 5b

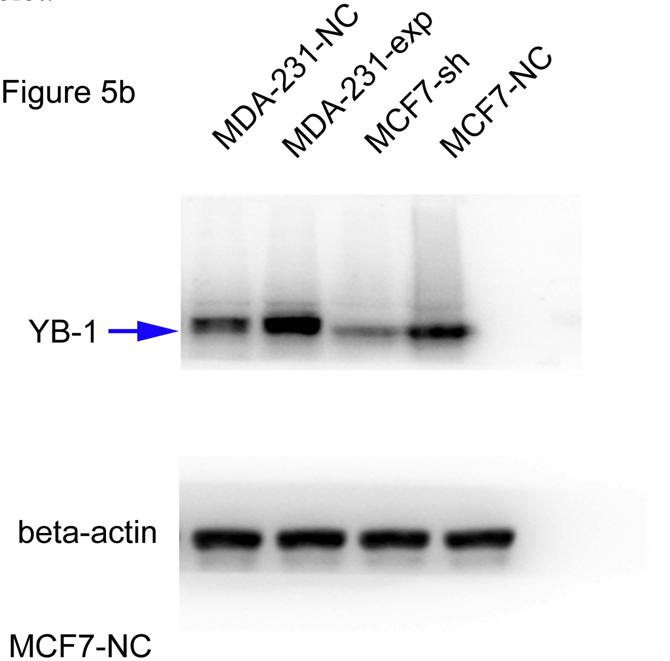

Figure 5c

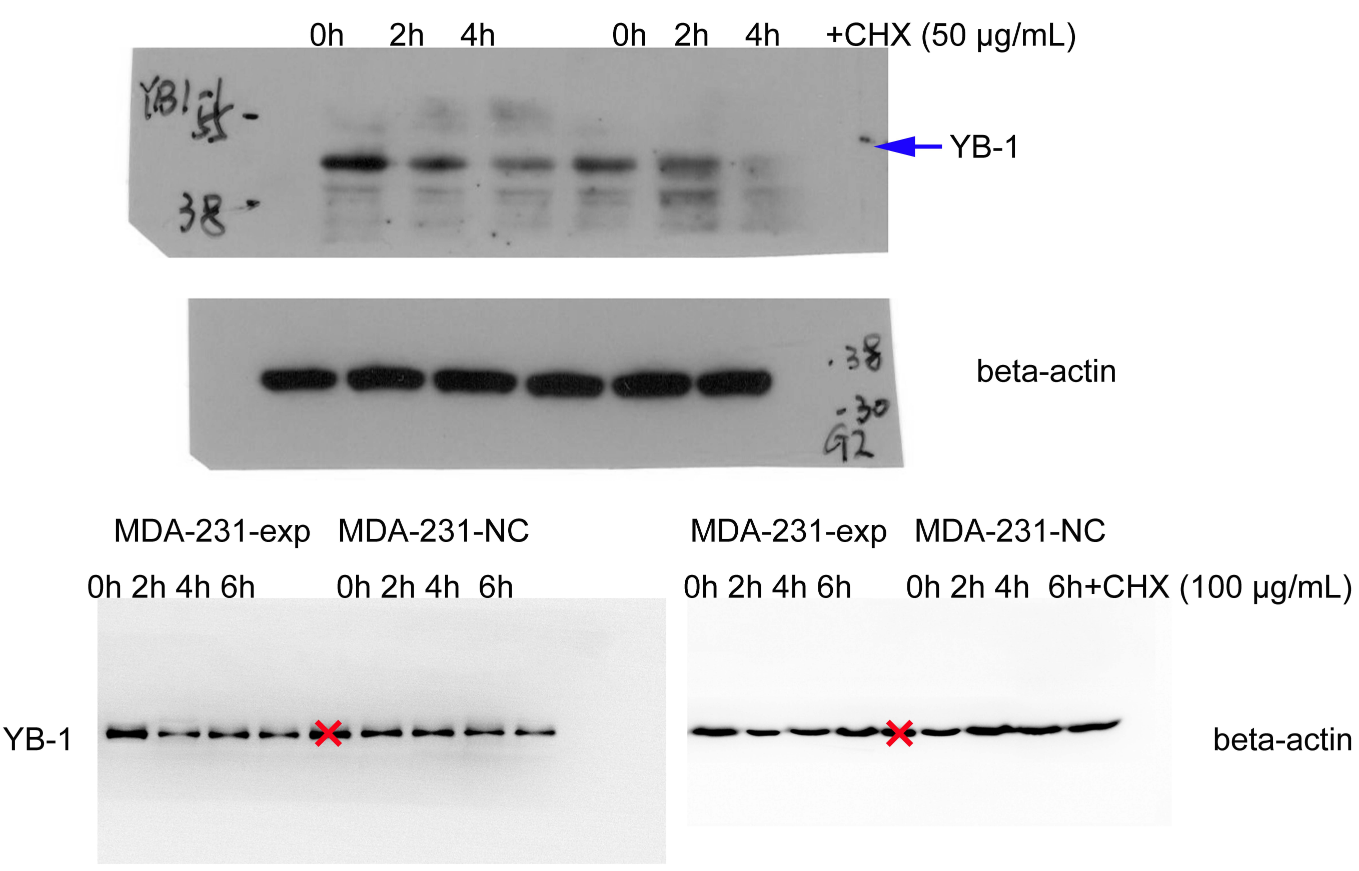

Figure 5d

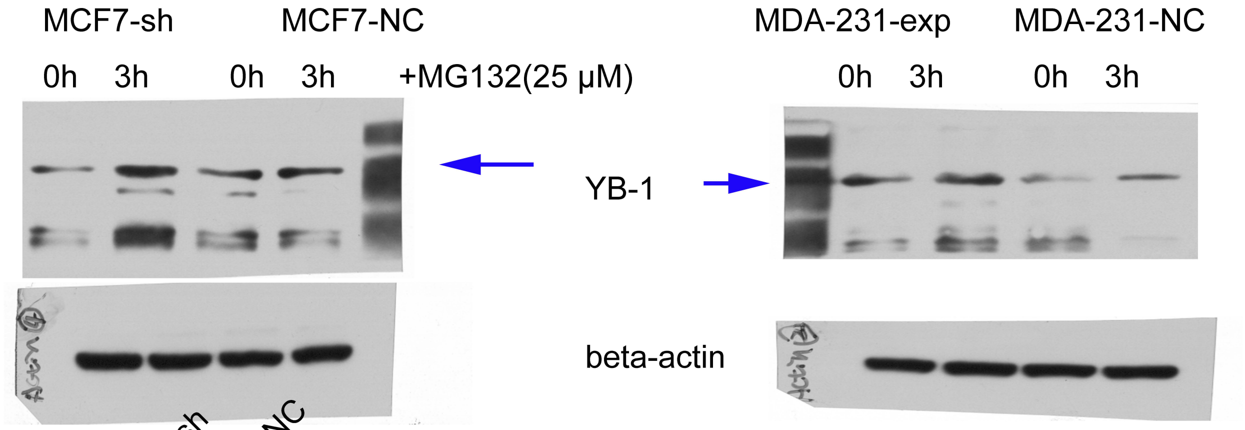

Figure 5e

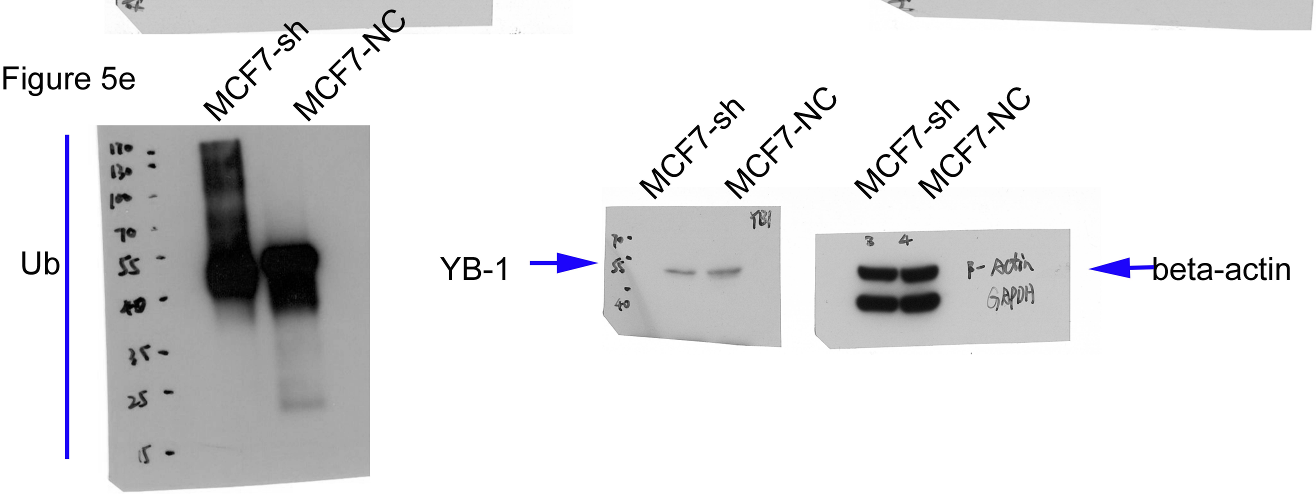

Figure 5f

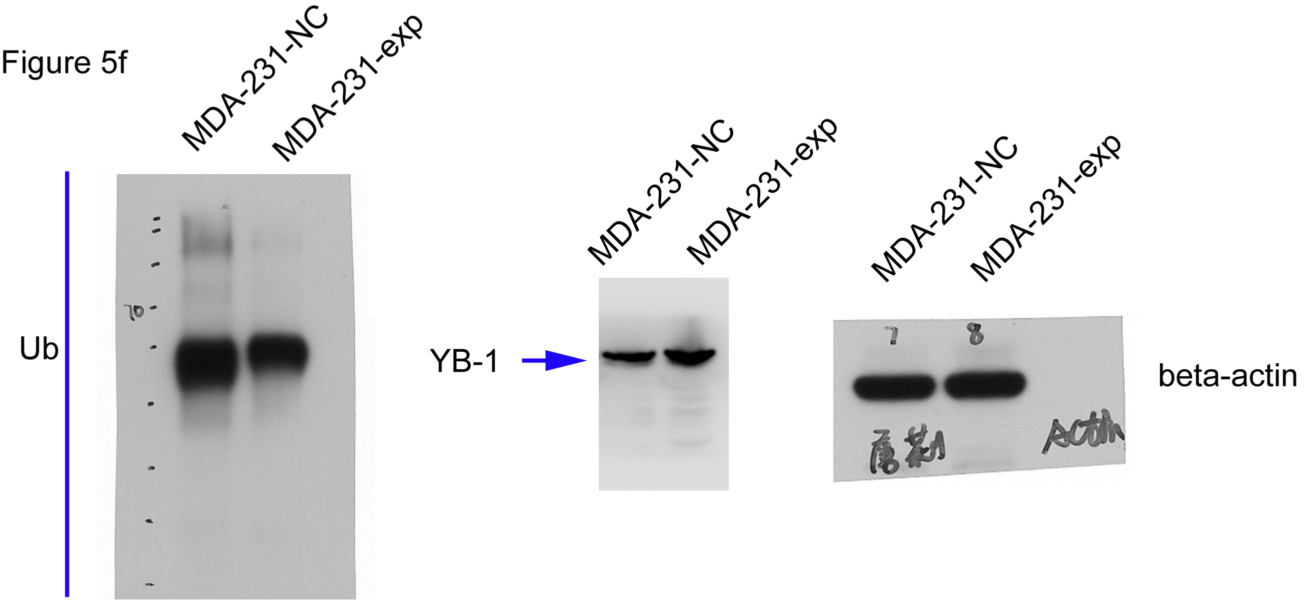

Figure 6b

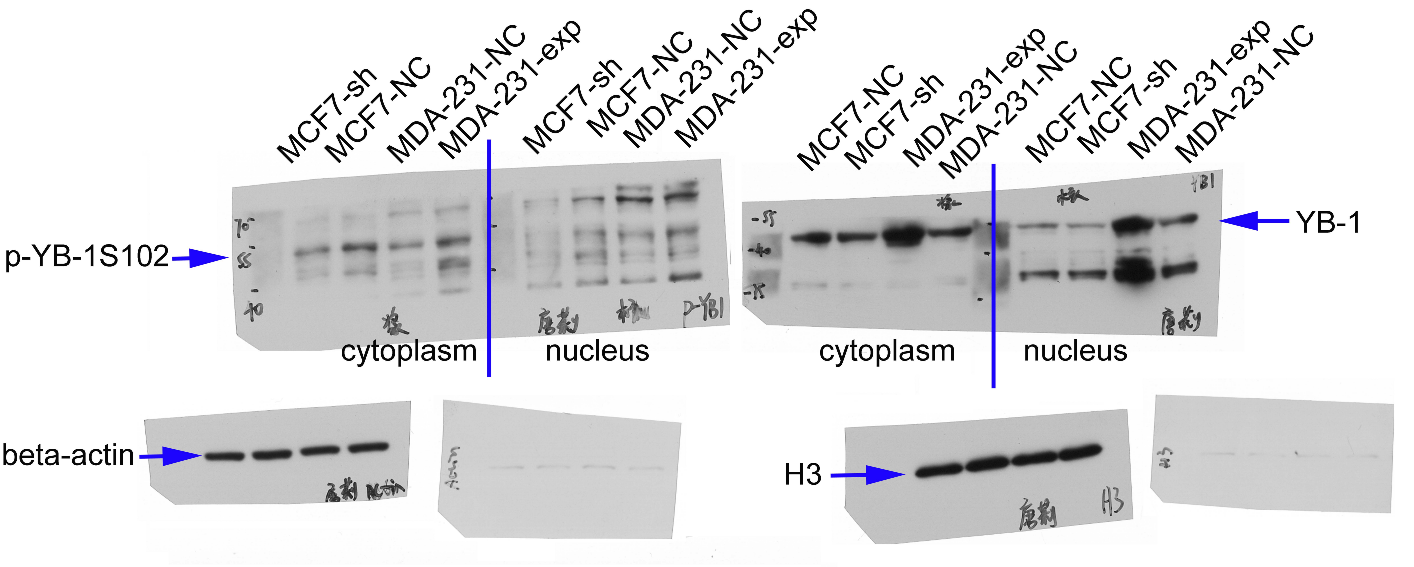

Figure 6c and 6d

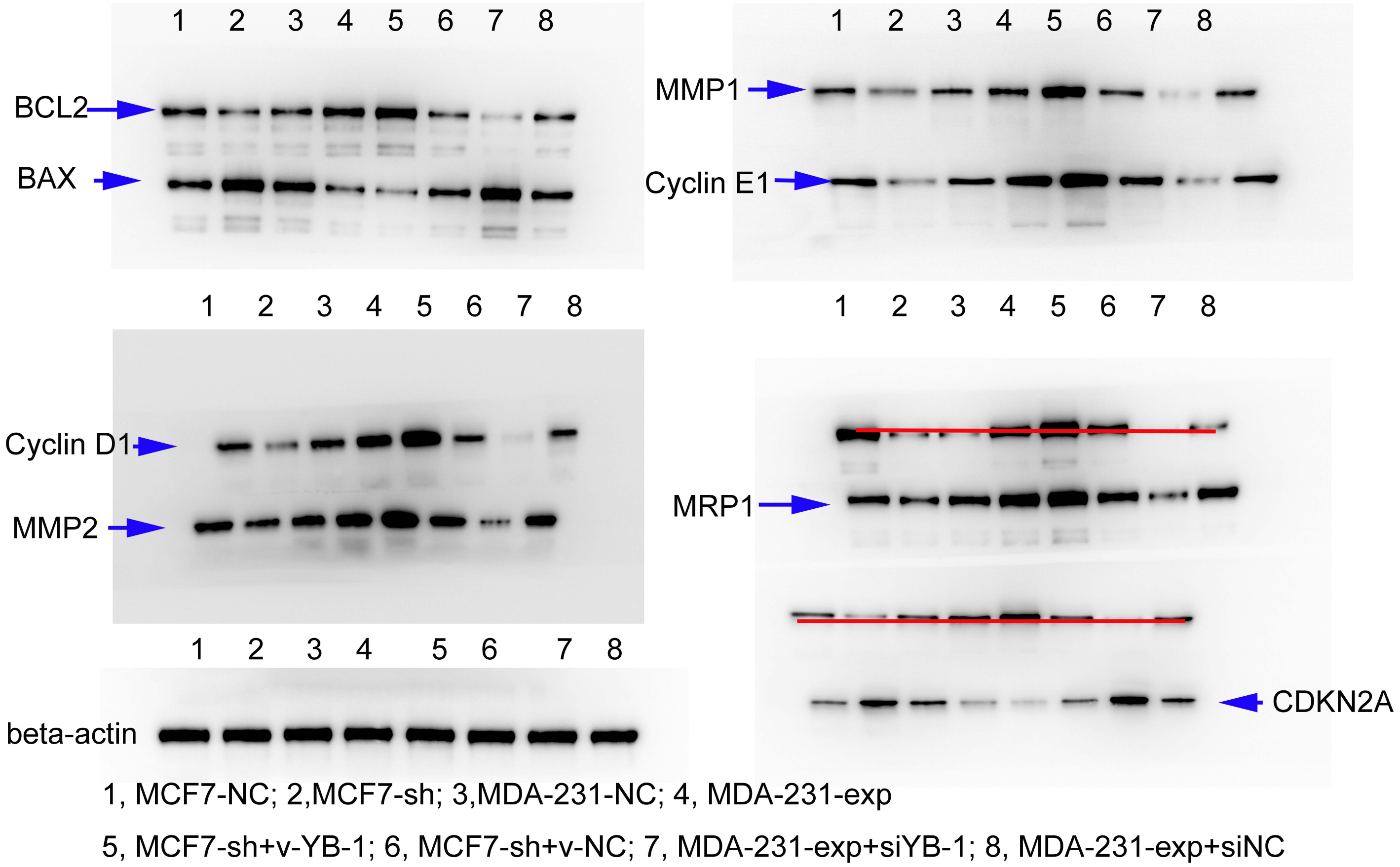

1, MCF7-NC; 2,MCF7-sh; 3,MDA-231-NC; 4, MDA-231-exp

5, MCF7-sh+v-YB-1; 6, MCF7-sh+v-NC; 7, MDA-231-exp+siYB-1; 8, MDA-231-exp+siNC

Supplementary note 1:

The antibodies used in this study.

Antibody	Specificity	Manufacturer	Code
anti-beta-actin	Rabbit polyclonal antibody	Abcam	ab8227
anti-histone H3	Rabbit polyclonal antibody	Abcam	ab1791
anti-CDKN2A	Rabbit monoclonal antibody	Abcam	ab108349
anti-P-gp	Rabbit monoclonal antibody	Abcam	ab170904
anti-MMP1	Rabbit monoclonal antibody	Abcam	ab52631
anti-MMP2	Rabbit monoclonal antibody	Abcam	ab92536
anti-cyclin D1	Rabbit monoclonal antibody	Abcam	ab40754
anti-cyclin E1	Rabbit monoclonal antibody	Abcam	ab33911
anti-BCL2	Rabbit monoclonal antibody	Abcam	ab32124
anti-BAX	Rabbit monoclonal antibody	Abcam	ab32503
anti-Ubiquitin	Rabbit monoclonal antibody	Abcam	ab134953
anti-YB-1	Rabbit monoclonal antibody	Abcam	ab76149
p-YB-1Ser102	Rabbit monoclonal antibody	CST	2900

Supplementary note 2:

Primer sequences used in quantitative PCR:

MIR200CHG Forward: 5′- GGAGGACTACTGACCAACAA-3′

Reverse: 5′- GGGAAGACAATGGAGGTG-3′

YB-1 Forward: 5′- GGTCCTCCACGCAATTACCA-3′

Reverse: 5′- GTTGTCAGCACCCTCCATCA-3′

GAPDH Forward: 5'-CCGGGAAACTGTGGCGTGATGG-3'

Reverse: 5'-AGGTGGAGGAGTGGGTGTCGCTGTT-3'

Primer sequences used in RNA pulldown:

MIR200CHG sense strands were amplified with the following primer sequence:

Rorward:

5'- TAATACGACTCACTATAGGGAAGAGCGGAGGCCAGGGCGGGCTCTAGGCC -3';

Reverse:

5'- TTTTTTTTTTTTTTTTTGACTTGGATCAGTCTTCC-3'.

MIR200CHG antisense strands amplified with the following primer sequences:

Forward:

5'-TAATACGACTCACTATAGGGTTTTTTTTTTTTTTTTTGACTTGGATCAGTCTTCC -3';

Reverse:

5'- AAGAGCGGAGGCCAGGGCGGGCTCTAGGCC -3'.

Supplementary note 3:

Lentiviral vector sequence that effectively interferes with MIR200CHG:

ATTACAAAAACAAATTACAAAAATTCAAAATTTTCGGGTTTATTACAGGGACAGCAG
AGATCCAGTTTGGTTAATTAATCGAGCGGCCGCCCCCTTCACCGAGGGCCTATTTCCC
ATGATTCCTTCATATTTGCATATACGATACAAGGCTGTTAGAGAGATAATTGGAATTA
ATTTGACTGTAAACACAAAGATATTAGTACAAAATACGTGACGTAGAAAGTAATAAT
TTCTTGGGTAGTTTGCAGTTTTAAAATTATGTTTTAAAATGGACTATCATATGCTTACC
GTAACTTGAAAGTATTTCGATTTCTTGGCTTTATATATCTTGTGGAAAGGACGAAACA
CCGGGAACATGAGGAAGACTGATCTCGAGATCAGTCTTCCTCATGTTCTTTTTGAATT
CTCGACCTCGAGACAAATGGCAGTATTCATCCACGGATCCTAACCCGTGTCGGCTCCA
GATCTGGCCTCCGCGCCGGGTTTTGGCGCCTCCCGCGGGCGCCCCCCTCCTCACGGCG
AGCGCTGCCACGTCAGACGAAGGGCGCAGCGAGCGTCCTGATCCTTCCGCCCGGACG
CTCAGGACAGCGGCCCGCTGCTCATAAGACTCGGCCTTAGAACCCCAGTATCAGCAG
AAGGACATTTTAGGACGGGACTTGGGTGACTCTAGGGCACTGGTTTTCTTTCCAGAGA
GCGGAACAGGCGAGGAAAAGTAGTCCCTTCTCGGCGATTCTGCGGAGGGATCTCCGT
GGGGCGGTGAACGCCGATGATTATATAAGGACGCGCCGGGTGTGGCACAGCTAGTTC
CGTCGCAGCCGGGATTTGGGGTCGCGGTTCTTGTTTGTGGATCGCTGTGATCGTCACT
TGTGAGTAGCGGGCTGCTGGGCTGCCGGGGCTTTCGTGCCGCCGGCCGCTCGGTGGG
ACGGAAGCGTGTGAGAAGACGCCAAGGGCTGTAGTCTGGGTCCGCGAGCAGTGCCTG
AACTGGGG

Synthetic oligo information:

NO.	5'	STEM	Loop	STEM	3'
LOC105369635-RNAi(70166-2)	Ccgg	GAACATGAGGAAGACTGAT	CTCGAG	ATCAGTCTTCCTCATGTTC	TTTTTg

YB-1siRNA and its negative control sequence:

siYB-1: GCAGACCGUAACCAUUAUATT

siNC: UUCUCCGAACGUGUCACGUTT

```
                                                     10        20        30        40        50        60        70        80
                                           CTTCGTATAGCATACATTATACGAAGTTATATTAAGGGTTCCAAGCTTAAGCGGCCGCAAGAGCGGAGGCCAGGGCGGGC
A4596-1_PEF-F_F08.ab1(50>500)        →
```

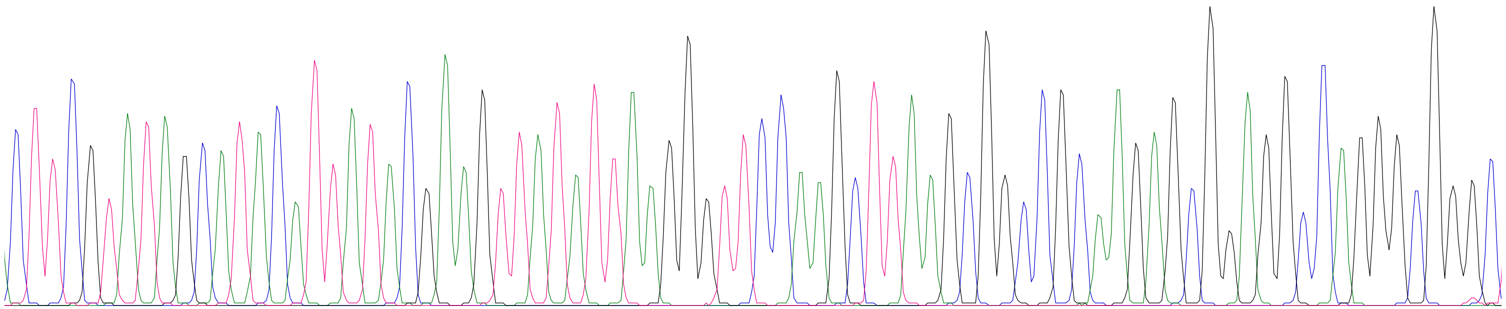

```
                                           CTTCGTATAGCATACATTATACGAAGTTATATTAAGGGTTCCAAGCTTAAGCGGCCGCAAGAGCGGAGGCCAGGGCGGGC
B4856 MIR200CHG homo.seq(1>366)       →                                                            AAGAGCGGAGGCCAGGGCGGGC
```

```
                                                     90       100       110       120       130       140       150       160
                                           TCTAGGCCGTGGAATCTGGGGCCTTAAAGCCCCTTCGTCTCCCCAGCACCCACTCTCTGGGGGCAGGTGGGCCCGGTGAC
A4596-1_PEF-F_F08.ab1(50>500)        →
```

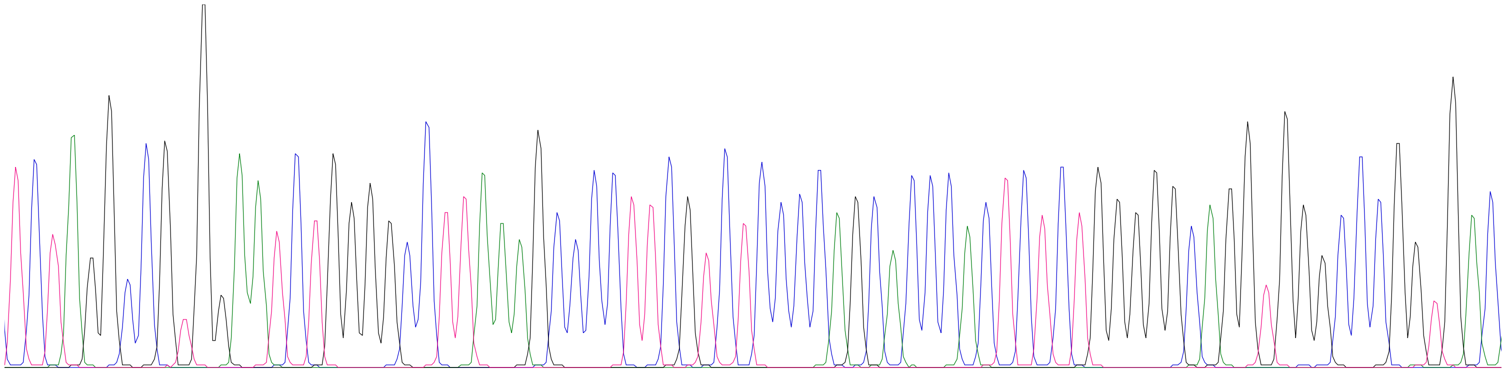

```
                                           TCTAGGCCGTGGAATCTGGGGCCTTAAAGCCCCTTCGTCTCCCCAGCACCCACTCTCTGGGGGCAGGTGGGCCCGGTGAC
B4856 MIR200CHG homo.seq(1>366)       →    TCTAGGCCGTGGAATCTGGGGCCTTAAAGCCCCTTCGTCTCCCCAGCACCCACTCTCTGGGGGCAGGTGGGCCCGGTGAC
```

```
                                                    170       180       190       200       210       220       230       240
                                           AGTAACCTTCAGGGAGCCCTGAAGACCATGGAGGACTACTGACCAACAACCTCTGACCTTCACCCCTCTGGATGGGGGAC
```

```
                                         170       180       190       200       210       220       230       240
                                 AGTAACCTTCAGGGAGCCCTGAAGACCATGGAGGACTACTGACCAACAACCTCTGACCTTCACCCCTCTGGATGGGGGAC
A4596-1_PEF-F_F08.ab1(50>500)  →
```

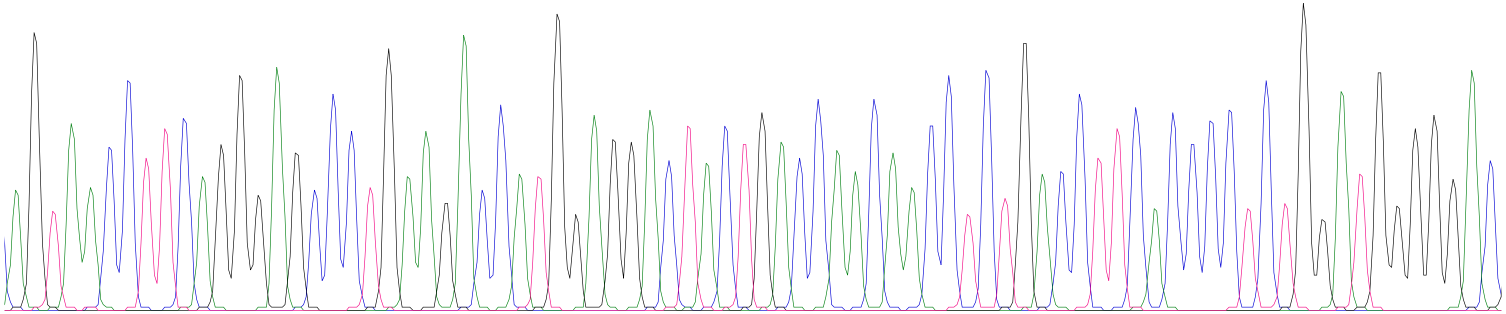

```
                                 AGTAACCTTCAGGGAGCCCTGAAGACCATGGAGGACTACTGACCAACAACCTCTGACCTTCACCCCTCTGGATGGGGGAC
B4856 MIR200CHG homo.seq(1>366) →  AGTAACCTTCAGGGAGCCCTGAAGACCATGGAGGACTACTGACCAACAACCTCTGACCTTCACCCCTCTGGATGGGGGAC

                                         250       260       270       280       290       300       310       320
                                 GAATCACTAGGCAAAGGGGAACAATGGGAAGGAGACAAAATGGCTGCCTTTACAGCTGCAGCAAGATGTGGAAACACTGG
A4596-1_PEF-F_F08.ab1(50>500)  →
```

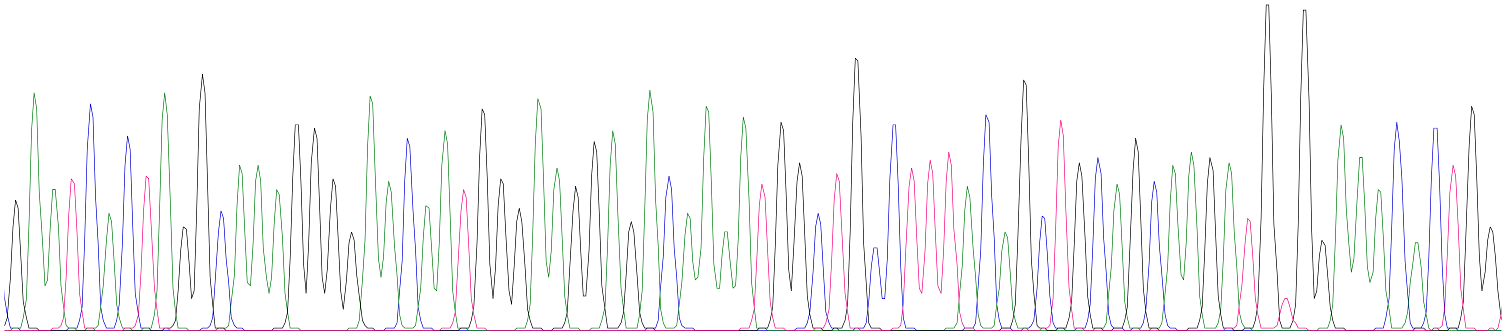

```
                                 GAATCACTAGGCAAAGGGGAACAATGGGAAGGAGACAAAATGGCTGCCTTTACAGCTGCAGCAAGATGTGGAAACACTGG
B4856 MIR200CHG homo.seq(1>366) →  GAATCACTAGGCAAAGGGGAACAATGGGAAGGAGACAAAATGGCTGCCTTTACAGCTGCAGCAAGATGTGGAAACACTGG

                                         330       340       350       360       370       380       390       400
                                 TTCCCTAGGCACCTCCATTGTCTTCCCGCCTTGGGAGCATGAAATAAATAAAGTGTGAAGGGGAACATGAGGAAGACTGA
```

```
                                 330       340       350       360       370       380       390       400
                              TTCCCTAGGCACCTCCATTGTCTTCCCGCCTTGGGAGCATGAAATAAATAAAGTGTGAAGGGGAACATGAGGAAGACTGA
A4596-1_PEF-F_F08.ab1(50>500) →
```

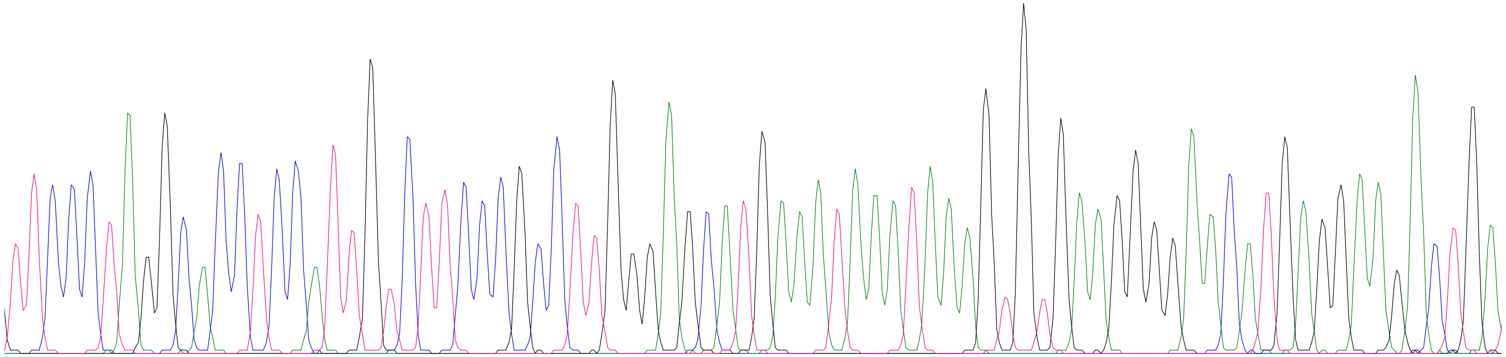

```
                              TTCCCTAGGCACCTCCATTGTCTTCCCGCCTTGGGAGCATGAAATAAATAAAGTGTGAAGGGGAACATGAGGAAGACTGA
B4856 MIR200CHG homo.seq(1>366) → TTCCCTAGGCACCTCCATTGTCTTCCCGCCTTGGGAGCATGAAATAAATAAAGTGTGAAGGGGAACATGAGGAAGACTGA

                                 410       420       430       440       450       460       470
                              TCCAAGTCAAAAAAAAAAAAAAAAAGGATCCGACACTCTCTACTGATACTCATCACTAACGTTACTGACATAACG
A4596-1_PEF-F_F08.ab1(50>500) →
```

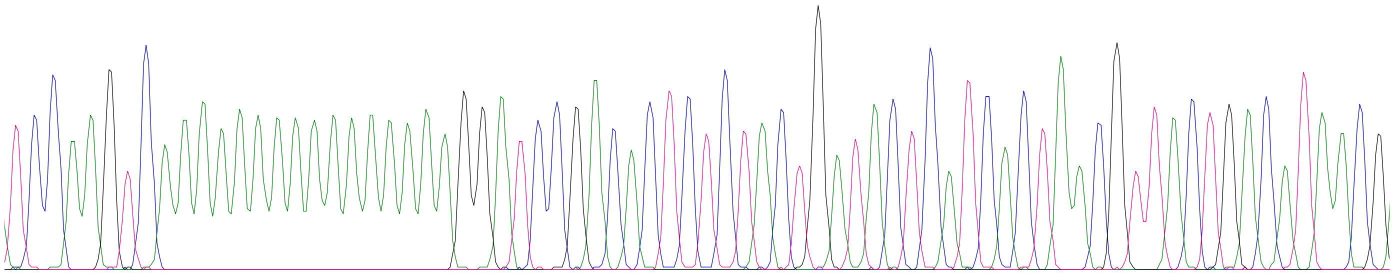

```
                              TCCAAGTCAAAAAAAAAAAAAAAAAGGATCCGACACTCTCTACTGATACTCATCACTAACGTTACTGACATAACG
B4856 MIR200CHG homo.seq(1>366) → TCCAAGTCAAAAAAAAAAAAAAAA
```

Supplementary Note 5: YB-1 expression vector sequence.

```
                10        20        30        40        50        60        70
ACTGGCTTATCGAAATTAATACGACTCACTATAGGGAGACCCAAGCTGGCTAGCGCCACCATGAGCAGCGAGGCC
```

V8034_1-CMV_F_D09.ab1(50>600) →

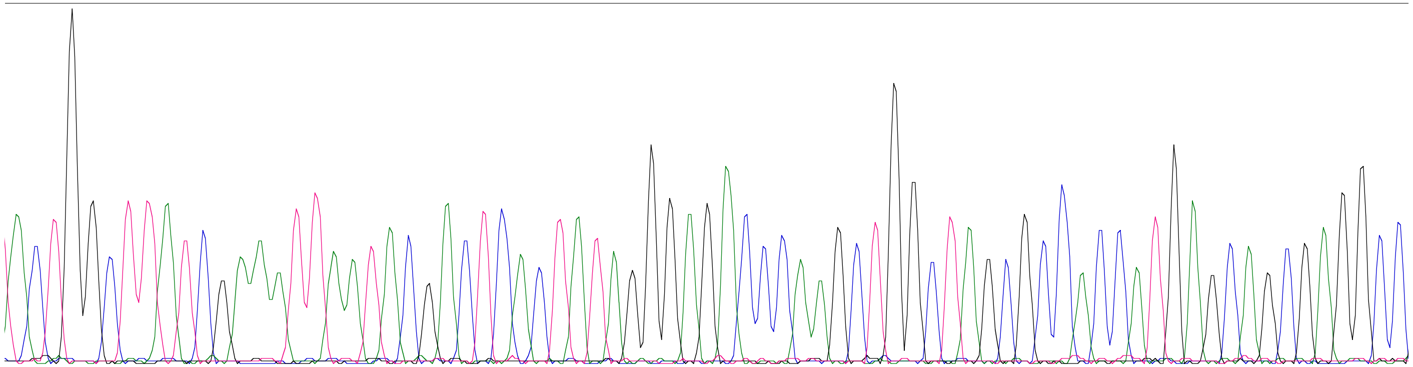

```
ACTGGCTTATCGAAATTAATACGACTCACTATAGGGAGACCCAAGCTGGCTAGCGCCACCATGAGCAGCGAGGCC
```

B2068 YBX1. NM_004559.3）.seq(1>975) →

```
                                                            atgagcagcgaggcc
```

```
      80        90        100       110       120       130       140       150
GAGACCCAGCAGCCGCCCGCCGCCCCCCCGCCGCCCCCGCCCTCAGCGCCGCCGACACCAAGCCCGGCACTACG
```

V8034_1-CMV_F_D09.ab1(50>600) →

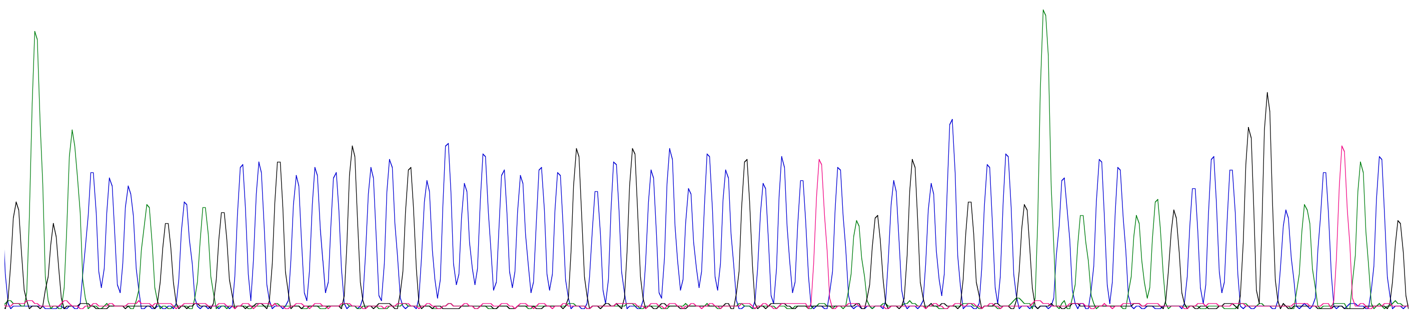

```
GAGACCCAGCAGCCGCCCGCCGCCCCCCCGCCGCCCCCGCCCTCAGCGCCGCCGACACCAAGCCCGGCACTACG
```

B2068 YBX1. NM_004559.3）.seq(1>975) →

```
gagacccagcagccgcccgccgcccccccgccgcccccgccctcagcgccgccgacaccaagcccggcactacg
```

```
           160       170       180       190       200       210       220
GGCAGCGGCGCAGGGAGCGGTGGCCCGGGCGGCCTCACATCGGCGGCGCCTGCCGGCGGGGACAAGAAGGTCATC
```

160 170 180 190 200 210 220

GGCAGCGGCGCAGGGAGCGGTGGCCCGGGCGGCCTCACATCGGCGGCGCCTGCCGGCGGGGACAAGAAGGTCATC

V8034_1-CMV_F_D09.ab1(50>600) →

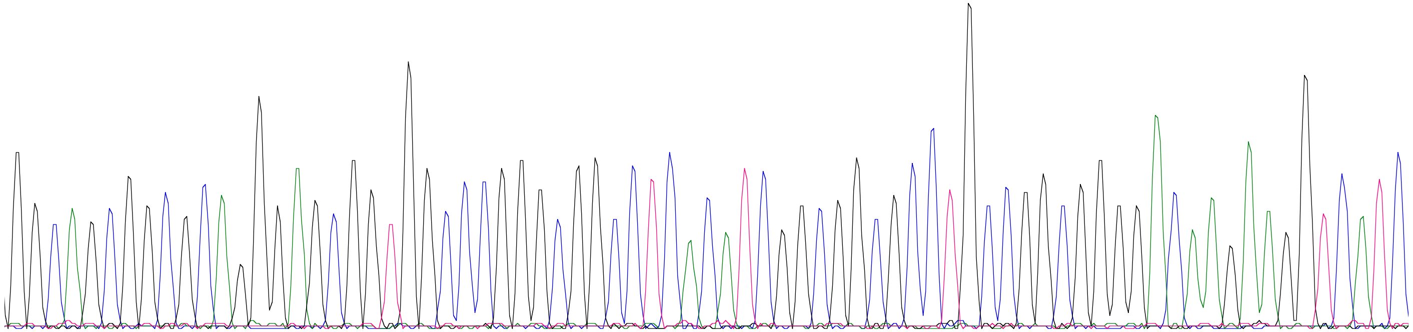

GGCAGCGGCGCAGGGAGCGGTGGCCCGGGCGGCCTCACATCGGCGGCGCCTGCCGGCGGGGACAAGAAGGTCATC

B2068 YBX1. NM_004559.3）.seq(1>975) → ggcagcggcgcagggagcggtggcccgggcggcctcacatcggcggcgcctgccggcggggacaagaaggtcatc

230 240 250 260 270 280 290 300

GCAACGAAGGTTTTGGGAACAGTAAAATGGTTCAATGTAAGGAACGGATATGGTTTCATCAACAGGAATGACACC

V8034_1-CMV_F_D09.ab1(50>600) →

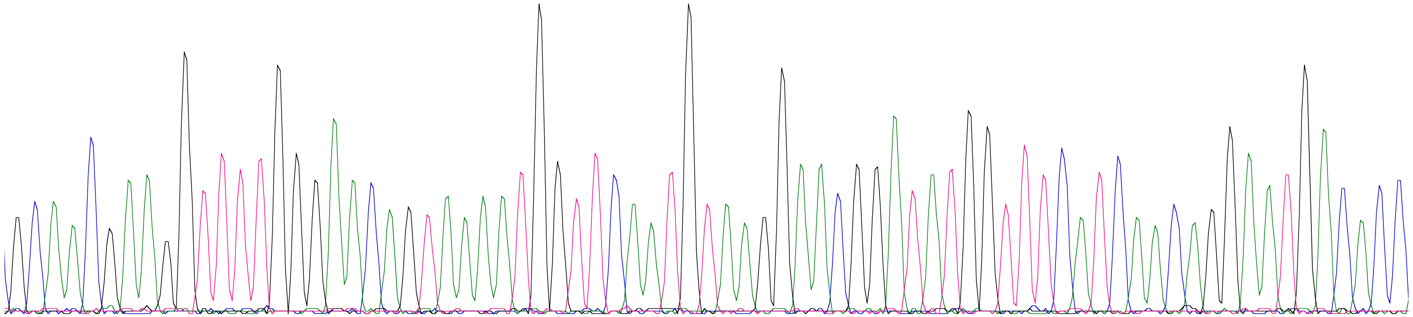

GCAACGAAGGTTTTGGGAACAGTAAAATGGTTCAATGTAAGGAACGGATATGGTTTCATCAACAGGAATGACACC

B2068 YBX1. NM_004559.3）.seq(1>975) → gcaacgaaggttttgggaacagtaaaatggttcaatgtaaggaacggatatggtttcatcaacaggaatgacacc

310 320 330 340 350 360 370

AAGGAAGATGTATTTGTACACCAGACTGCCATAAAGAAGAATAACCCCAGGAAGTACCTTCGCAGTGTAGGAGAT

```
                                          310       320       330       340       350       360       370
                                  AAGGAAGATGTATTTGTACACCAGACTGCCATAAAGAAGAATAACCCCAGGAAGTACCTTCGCAGTGTAGGAGAT
V8034_1-CMV_F_D09.ab1(50>600)  →
```

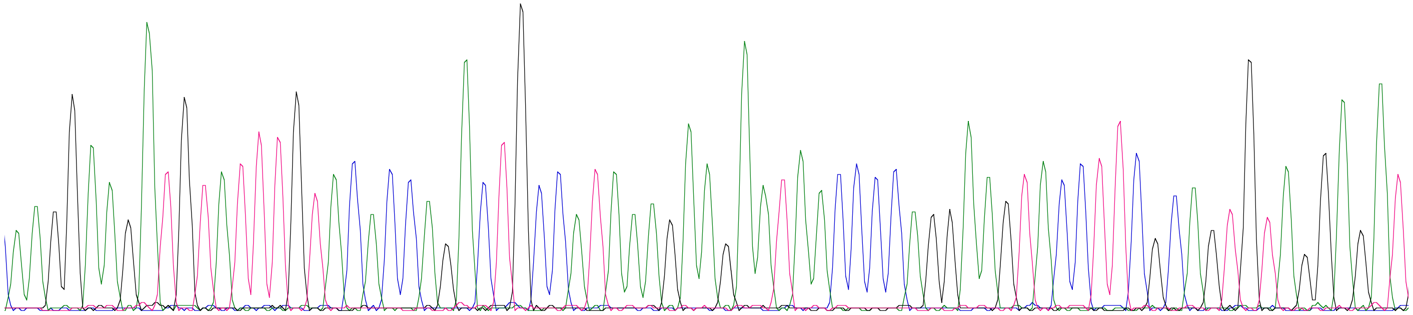

```
                                  AAGGAAGATGTATTTGTACACCAGACTGCCATAAAGAAGAATAACCCCAGGAAGTACCTTCGCAGTGTAGGAGAT
B2068 YBX1. NM_004559.3）.seq(1>975)  →  aaggaagatgtatttgtacaccagactgccataaagaagaataaccccaggaagtaccttcgcagtgtaggagat

                                      380       390       400       410       420       430       440       450
                                  GGAGAGACTGTGGAGTTTGATGTTGTTGAAGGAGAAAAGGGTGCGGAGGCAGCAAATGTTACAGGTCCTGGTGGT
V8034_1-CMV_F_D09.ab1(50>600)  →
```

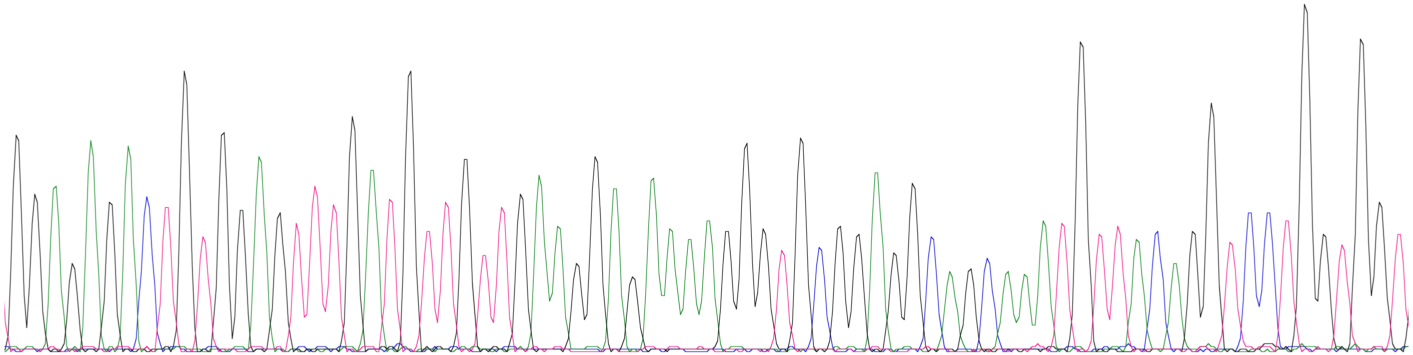

```
                                  GGAGAGACTGTGGAGTTTGATGTTGTTGAAGGAGAAAAGGGTGCGGAGGCAGCAAATGTTACAGGTCCTGGTGGT
B2068 YBX1. NM_004559.3）.seq(1>975)  →  ggagagactgtggagtttgatgttgttgaaggagaaaagggtgcggaggcagcaaatgttacaggtcctggtggt

                                          460       470       480       490       500       510       520
                                  GTTCCAGTTCAAGGCAGTAAATATGCAGCAGACCGTAACCATTATAGACGCTATCCACGTCGTAGGGGTCCTCCA
```

```
                                               460       470       480       490       500       510       520
                                             GTTCCAGTTCAAGGCAGTAAATATGCAGCAGACCGTAACCATTATAGACGCTATCCACGTCGTAGGGGTCCTCCA
V8034_1-CMV_F_D09.ab1(50>600)            →
```

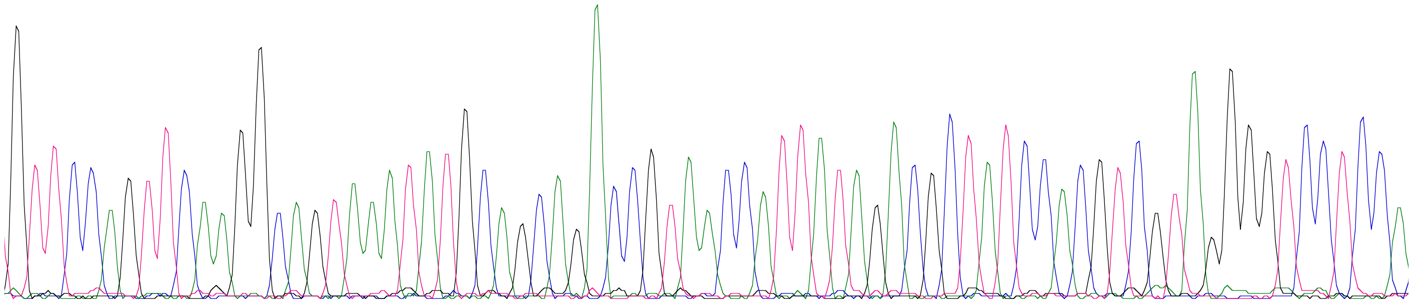

```
                                             GTTCCAGTTCAAGGCAGTAAATATGCAGCAGACCGTAACCATTATAGACGCTATCCACGTCGTAGGGGTCCTCCA
B2068 YBX1. NM_004559.3）.seq(1>975)     →  gttccagttcaaggcagtaaatatgcagcagaccgtaaccattatagacgctatccacgtcgtaggggtcctcca
V8034_1-BGH_E09.ab1(50>600)              ←
```

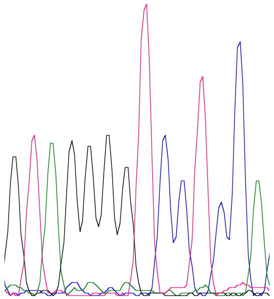

```
                                                                                                GTAGGGGTCCTCCA

                                              530       540       550       560       570       580       590       600
                                             CGCAATTACCAGCAAAATTACCAGAATAGTGAGAGTGGGGAAAAGAACGAGGGATCGGAGAGTGCTCCCGAAGGC
```

```
                                  530       540       550       560       570       580       590       600
                                  CGCAATTACCAGCAAAATTACCAGAATAGTGAGAGTGGGGAAAAGAACGAGGGATCGGAGAGTGCTCCCGAAGGC
V8034_1-CMV_F_D09.ab1(50>600)  →
```

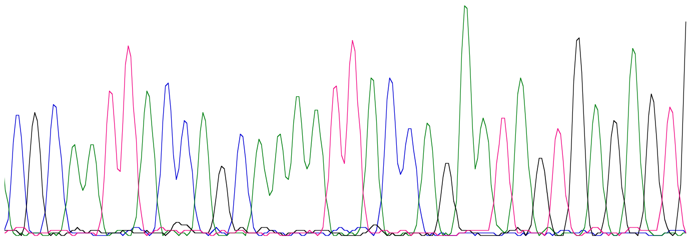

```
                                  CGCAATTACCAGCAAAATTACCAGAATAGTGAGAGT
B2068 YBX1. NM_004559.3）.seq(1>975) →  cgcaattaccagcaaaattaccagaatagtgagagtggggaaaagaacgagggatcggagagtgctcccgaaggc
V8034_1-BGH_E09.ab1(50>600)    ←
```

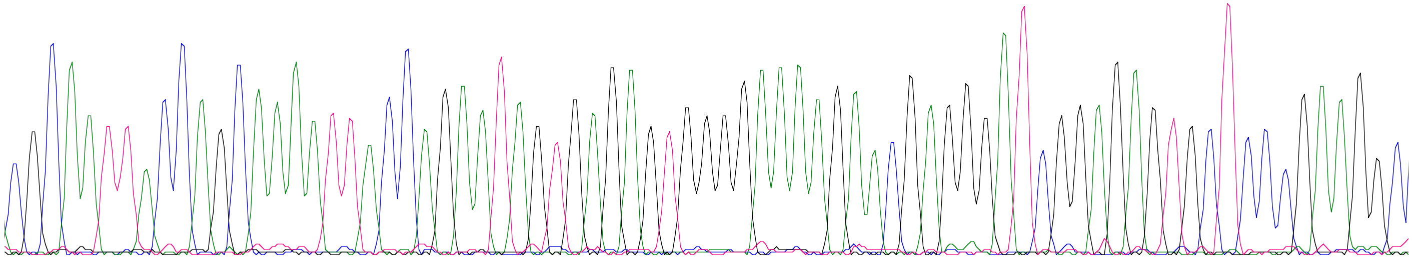

```
                                  CGCAATTACCAGCAAAATTACCAGAATAGTGAGAGTGGGGAAAAGAACGAGGGATCGGAGAGTGCTCCCGAAGGC
```

```
                                                               610       620       630       640       650       660       670
                                                          CAGGCCCAACAACGCCGGCCCTACCGCAGGCGAAGGTTCCCACCTTACTACATGCGGAGACCCTATGGGCGTCGA
B2068 YBX1. NM_004559.3）.seq(1>975)          →          caggcccaacaacgccggccctaccgcaggcgaaggttcccaccttactacatgcggagaccctatgggcgtcga
V8034_1-BGH_E09.ab1(50>600)                   ←
```

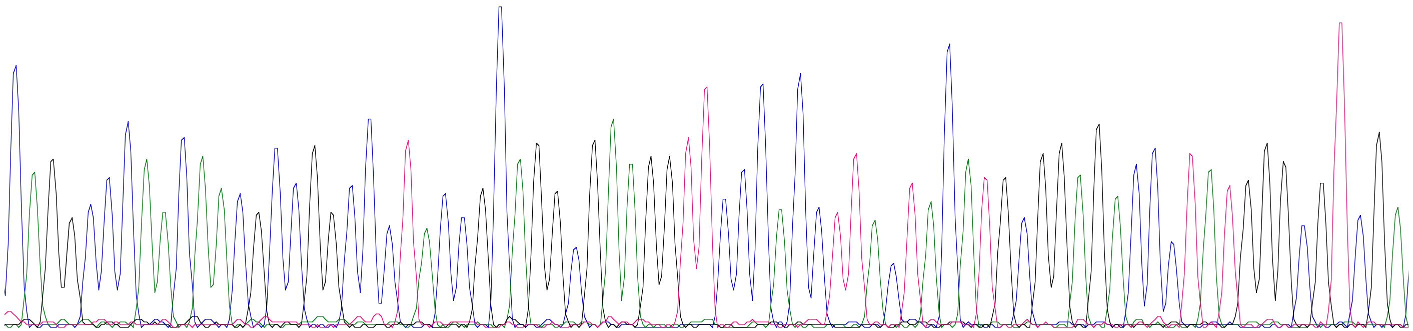

```
                                                          CAGGCCCAACAACGCCGGCCCTACCGCAGGCGAAGGTTCCCACCTTACTACATGCGGAGACCCTATGGGCGTCGA
```

```
                                                          680       690       700       710       720       730       740       750
                                                          CCACAGTATTCCAACCCTCCTGTGCAGGGAGAAGTGATGGAGGGTGCTGACAACCAGGGTGCAGGAGAACAAGGT
B2068 YBX1. NM_004559.3）.seq(1>975)          →          ccacagtattccaaccctcctgtgcagggagaagtgatggagggtgctgacaaccagggtgcaggagaacaaggt
V8034_1-BGH_E09.ab1(50>600)                   ←
```

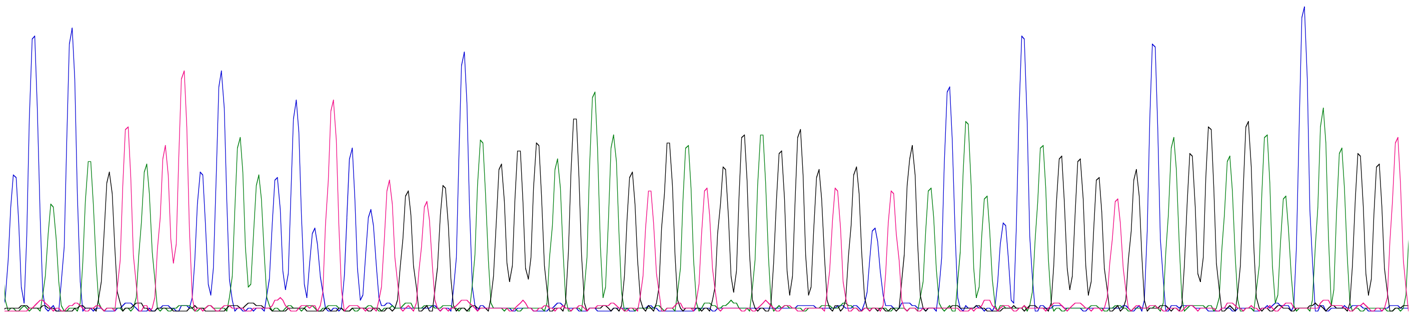

```
                                                          CCACAGTATTCCAACCCTCCTGTGCAGGGAGAAGTGATGGAGGGTGCTGACAACCAGGGTGCAGGAGAACAAGGT
```

760 770 780 790 800 810 820

AGACCAGTGAGGCAGAATATGTATCGGGGATATAGACCACGATTCCGCAGGGGCCCTCCTCGCCAAAGACAGCCT

B2068 YBX1．NM_004559.3）.seq(1>975) → agaccagtgaggcagaatatgtatcggggatatagaccacgattccgcaggggccctcctcgccaaagacagcct
V8034_1-BGH_E09.ab1(50>600) ←

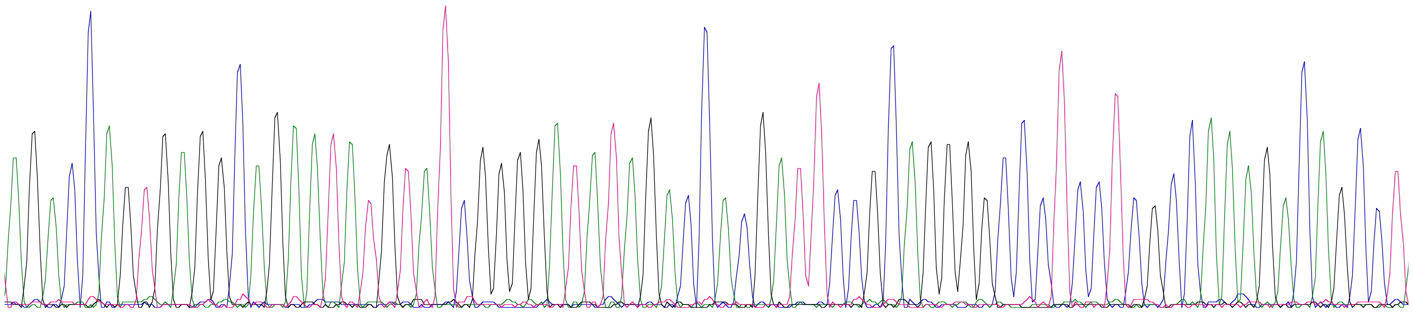

AGACCAGTGAGGCAGAATATGTATCGGGGATATAGACCACGATTCCGCAGGGGCCCTCCTCGCCAAAGACAGCCT

830 840 850 860 870 880 890 900

AGAGAGGACGGCAATGAAGAAGATAAAGAAAATCAAGGAGATGAGACCCAAGGTCAGCAGCCACCTCAACGTCGG

B2068 YBX1．NM_004559.3）.seq(1>975) → agagaggacggcaatgaagaagataaagaaaatcaaggagatgagacccaaggtcagcagccacctcaacgtcgg
V8034_1-BGH_E09.ab1(50>600) ←

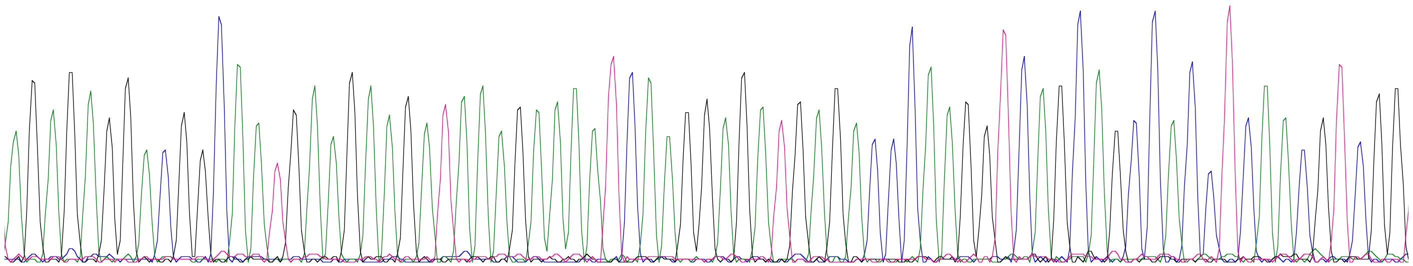

AGAGAGGACGGCAATGAAGAAGATAAAGAAAATCAAGGAGATGAGACCCAAGGTCAGCAGCCACCTCAACGTCGG

910 920 930 940 950 960 970

TACCGCCGCAACTTCAATTACCGACGCAGACGCCCAGAAAACCCTAAACCACAAGATGGCAAAGAGACAAAAGCA

B2068 YBX1. NM_004559.3）.seq(1>975) → taccgccgcaacttcaattaccgacgcagacgcccagaaaaccctaaaccacaagatggcaaagagacaaaagca
V8034_1-BGH_E09.ab1(50>600) ←

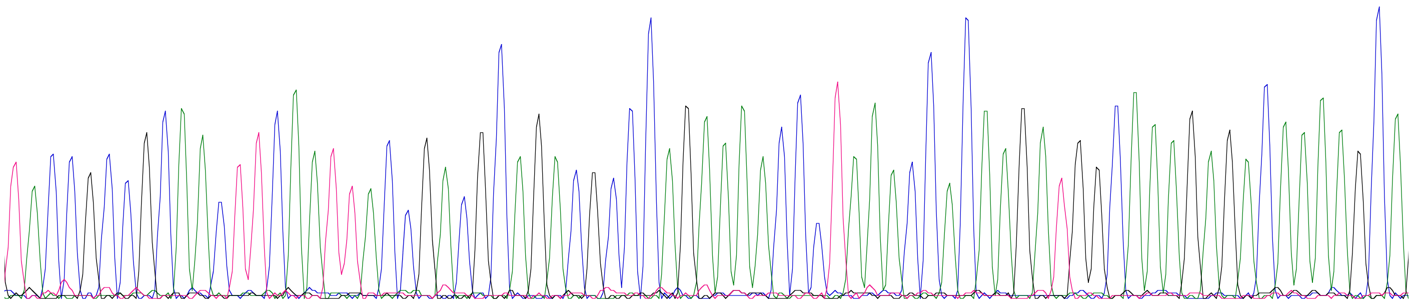

TACCGCCGCAACTTCAATTACCGACGCAGACGCCCAGAAAACCCTAAACCACAAGATGGCAAAGAGACAAAAGCA

980 990 1000 1010 1020 1030 1040 1050

GCCGATCCACCAGCTGAGAATTCGTCCGCTCCCGAGGCTGAGCAGGGCGGGGCTGAGTAAGGATCCACTAGTCCA

B2068 YBX1. NM_004559.3）.seq(1>975) → gccgatccaccagctgagaattcgtccgctcccgaggctgagcagggcggggctgagtaa
V8034_1-BGH_E09.ab1(50>600) ←

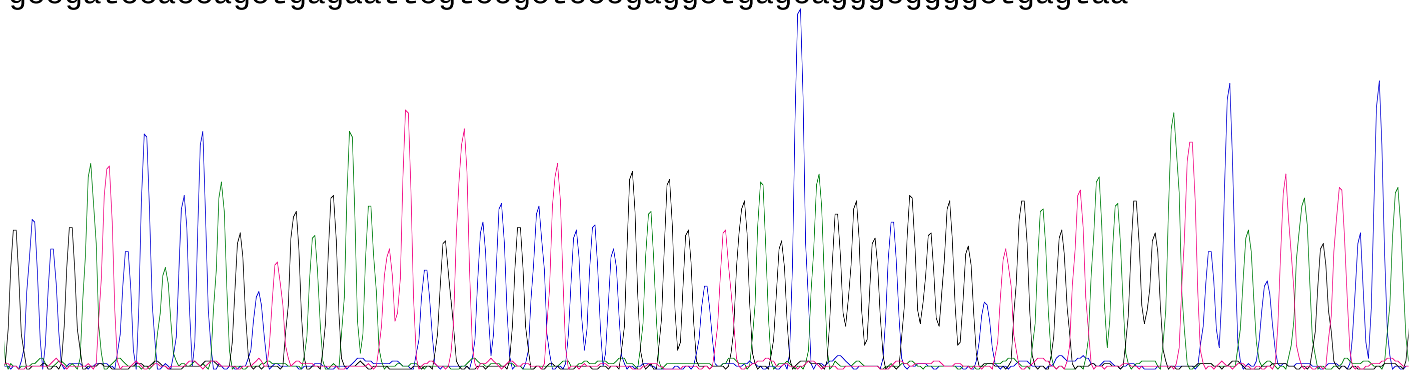

GCCGATCCACCAGCTGAGAATTCGTCCGCTCCCGAGGCTGAGCAGGGCGGGGCTGAGTAAGGATCCACTAGTCCA

1060 1070 1080 1090

GTGTGGTGGAATTCTGCAGATATCCAGCACAGTGGCGGCCGC

1060 1070 1080 1090

GTGTGGTGGAATTCTGCAGATATCCAGCACAGTGGCGGCCGC

V8034_1-BGH_E09.ab1(50>600) ←

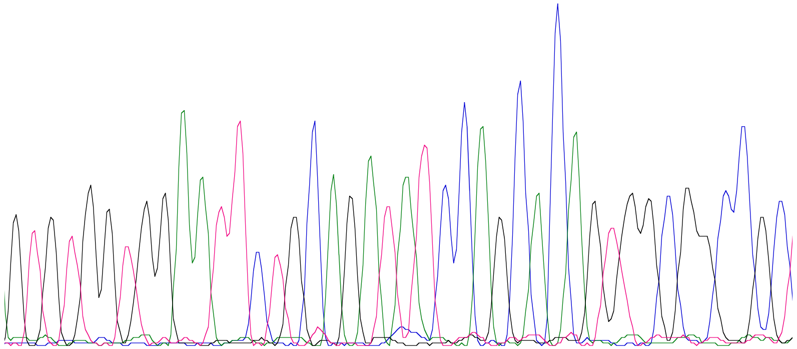

GTGTGGTGGAATTCTGCAGATATCCAGCACAGTGGCGGCCGC

Supplementary note 6:

MIR200CHG probe sequences used in FISH experiment.

Sequence name	Probe sequence (5’-3’)	Modification
NR_135032.1-1	gagacg+aaggggctttaag+g	5’-biotin
NR_135032.1-2	cat+ccaga+ggggtgaaggtc	5’-biotin
NR_135032.1-3	tcttgc+tgcagctgta+aagg	5’-biotin
