# Supplementary material for: Long non-coding RNA MIR200CHG promotes breast cancer proliferation, invasion, and drug resistance by interacting with and stabilizing YB-1
Source: NPJ Breast Cancer. 2021 Jul 16;7:94. doi: 10.1038/s41523-021-00293-x (PMC8285504; doi:10.1038/s41523-021-00293-x)
Supplement: Supplementary file 2 — Reporting Summary [file 41523_2021_293_MOESM2_ESM.pdf]

nature research

Corresponding author(s): Li Tang, Feng Yan

Last updated by author(s): Jun 5, 2021

Reporting Summary

Nature Research wishes to improve the reproducibility of the work that we publish. This form provides structure for consistency and transparency in reporting. For further information on Nature Research policies, see our Editorial Policies and the Editorial Policy Checklist.

Statistics

For all statistical analyses, confirm that the following items are present in the figure legend, table legend, main text, or Methods section.

n/a	Confirmed	
☐	☒	The exact sample size (*n*) for each experimental group/condition, given as a discrete number and unit of measurement
☐	☒	A statement on whether measurements were taken from distinct samples or whether the same sample was measured repeatedly
☐	☒	The statistical test(s) used AND whether they are one- or two-sided<br>*Only common tests should be described solely by name; describe more complex techniques in the Methods section.*
☐	☒	A description of all covariates tested
☐	☒	A description of any assumptions or corrections, such as tests of normality and adjustment for multiple comparisons
☐	☒	A full description of the statistical parameters including central tendency (e.g. means) or other basic estimates (e.g. regression coefficient) AND variation (e.g. standard deviation) or associated estimates of uncertainty (e.g. confidence intervals)
☐	☒	For null hypothesis testing, the test statistic (e.g. *F*, *t*, *r*) with confidence intervals, effect sizes, degrees of freedom and *P* value noted<br>*Give P values as exact values whenever suitable.*
☒	☐	For Bayesian analysis, information on the choice of priors and Markov chain Monte Carlo settings
☒	☐	For hierarchical and complex designs, identification of the appropriate level for tests and full reporting of outcomes
☐	☒	Estimates of effect sizes (e.g. Cohen's *d*, Pearson's *r*), indicating how they were calculated

Our web collection on statistics for biologists contains articles on many of the points above.

Software and code

Policy information about availability of computer code

Data collection	Microsoft Excel
Data analysis	R Project, GraphPad Prism 8 and SPSS version 19.0

For manuscripts utilizing custom algorithms or software that are central to the research but not yet described in published literature, software must be made available to editors and reviewers. We strongly encourage code deposition in a community repository (e.g. GitHub). See the Nature Research guidelines for submitting code & software for further information.

Data

Policy information about availability of data

All manuscripts must include a data availability statement. This statement should provide the following information, where applicable:

- Accession codes, unique identifiers, or web links for publicly available datasets
- A list of figures that have associated raw data
- A description of any restrictions on data availability

The data generated and analyzed during this study are described in the following data record: https://doi.org/10.6084/m9.figshare.14731272 . The lncRNA microarray data of breast cancer tissues have been deposited in NCBI Gene Expression Omnibus database (GEO) and are openly available via accession https://identifiers.org/geo:GSE115275 . The mass spectrometry results of RNA pull-down protein solutions have been deposited in PRIDE and are available via accession https://identifiers.org/pride.project:PXD026480 . The TCGA data used in this study is publicly available on the National Cancer Institute's (NCI) Genomic Data Commons (GDC) (https://portal.gdc.cancer.gov/). The search terms used to locate the data in GDC were: BRCA RNAseq: breast, TCGA-BRCA, transcriptome profiling, HTseq-FPKM; BRCA miRNAseq: breast, TCGA-BRCA, transcriptome profiling, Isoform Expression Quantification, miRNA-Seq, BCGSC miRNA Profiling.Under

reasonable circumstances, the materials generated in this study can be obtained from the corresponding authors. The raw data of all Western blots are provided in Supplementary Figure 5.

Field-specific reporting

Please select the one below that is the best fit for your research. If you are not sure, read the appropriate sections before making your selection.

☒ Life sciences ☐ Behavioural & social sciences ☐ Ecological, evolutionary & environmental sciences

For a reference copy of the document with all sections, see nature.com/documents/nr-reporting-summary-flat.pdf

Life sciences study design

All studies must disclose on these points even when the disclosure is negative.

Sample size	52
Data exclusions	N/A
Replication	All experiments were performed at least two or three times in triplicate.
Randomization	N/A
Blinding	N/A

Reporting for specific materials, systems and methods

We require information from authors about some types of materials, experimental systems and methods used in many studies. Here, indicate whether each material, system or method listed is relevant to your study. If you are not sure if a list item applies to your research, read the appropriate section before selecting a response.

Materials & experimental systems

n/a	Involved in the study	
☐	☒	Antibodies
☐	☒	Eukaryotic cell lines
☒	☐	Palaeontology and archaeology
☐	☒	Animals and other organisms
☒	☐	Human research participants
☒	☐	Clinical data
☒	☐	Dual use research of concern

Methods

n/a	Involved in the study	
☒	☐	ChIP-seq
☐	☒	Flow cytometry
☒	☐	MRI-based neuroimaging

Antibodies

Antibodies used	The antibodies purchased from Abcam (Cambridge, Massachusetts, USA) used in this study are anti-β-actin (ab8227), anti-histone H3 (ab1791), anti-CDKN2A (ab108349), anti-MRP1 (ab170904), and anti-MMP1 (ab52631), anti-MMP2 (ab92536), anti-cyclin D1 (ab40754), anti-cyclin E1 (ab33911), anti-BCL2 (ab32124), anti-BAX (ab32503), anti-ubiquitin (ab134953) and anti-YB-1 (ab76149). The antibody purchased from Cell Signaling Technology (Denver, Massachusetts, USA) used in this study was anti-pYB-1ser102 (2900).
Validation	All antibodies are commercially available and have been validated by the company or numerous research articles.

Eukaryotic cell lines

Policy information about cell lines

Cell line source(s)	Breast cancer cell lines MCF7, T-47D, BT549 and MDA-MB-231 were obtained from the Cell Bank of the Chinese Academy of Sciences (Shanghai, China) and cultured according to the ATCC recommended protocol.
Authentication	The cell lines were purchased directly from the Cell Bank of the Chinese Academy of Sciences without other verification.
Mycoplasma contamination	All the cells lines are Mycoplasma negative. Periodic tests have been performed using MycoProbe™ Mycoplasma Detection Kit (R&D Systems).
Commonly misidentified lines (See ICLAC register)	Not relevant to our study

Animals and other organisms

Policy information about studies involving animals; ARRIVE guidelines recommended for reporting animal research

Laboratory animals	BALB / c nude mice were purchased from Cavins Laboratory Animal Co., Ltd., and the strain and number of mice used in the experiment are mentioned in the manuscript.
Wild animals	Not relevant to our study
Field-collected samples	We used a digital caliper to measure the length (mm) and width (mm) of the tumor. The mice were sacrificed after 37 days, and the tumor tissues were excised and weighed.
Ethics oversight	All animal studies were approved by the Institutional Animal Care and Use Committee of Hubei Medical College of Preventive Sciences and conducted in accordance with the National Institutes of Health's Guide for the Care and Use of Laboratory Animals.

Note that full information on the approval of the study protocol must also be provided in the manuscript.

Flow Cytometry

Plots

Confirm that:

☒ The axis labels state the marker and fluorochrome used (e.g. CD4-FITC).

☒ The axis scales are clearly visible. Include numbers along axes only for bottom left plot of group (a 'group' is an analysis of identical markers).

☒ All plots are contour plots with outliers or pseudocolor plots.

☒ A numerical value for number of cells or percentage (with statistics) is provided.

Methodology

Sample preparation	PI staining and Annexin-V APC/7-AAD double staining were performed according to the manufacturer's instructions.
Instrument	FACSCalibur, BD Biosciences
Software	CellQuest software
Cell population abundance	All our analysis were from a homogeneous population (established breast cancer cell lines), containing at least 5000 single cells.
Gating strategy	*Describe the gating strategy used for all relevant experiments, specifying the preliminary FSC/SSC gates of the starting cell population, indicating where boundaries between "positive" and "negative" staining cell populations are defined.*

☐ Tick this box to confirm that a figure exemplifying the gating strategy is provided in the Supplementary Information.
